# Smart Soup, a Traditional Chinese Medicine Formula, Ameliorates Amyloid Pathology and Related Cognitive Deficits

**DOI:** 10.1371/journal.pone.0111215

**Published:** 2014-11-11

**Authors:** Yujun Hou, Ying Wang, Jian Zhao, Xiaohang Li, Jin Cui, Jianqing Ding, Ying Wang, Xianglu Zeng, Yun Ling, Xiaoheng Shen, Shengdi Chen, Chenggang Huang, Gang Pei

**Affiliations:** 1 State Key Laboratory of Cell Biology, Institute of Biochemistry and Cell Biology, Shanghai Institutes for Biological Sciences, Graduate School of the Chinese Academy of Sciences, Chinese Academy of Sciences, Shanghai, China; 2 Institute of Neurology, Ruijin Hospital, Shanghai Jiaotong University School of Medicine, Shanghai, China; 3 Department of Neurology, Ruijin Hospital, Shanghai Jiaotong University School of Medicine, Shanghai, China; 4 Shanghai Institute of Materia Medica, Chinese Academy of Sciences, Shanghai, China; 5 Department of Traditional Chinese Medicine, Ruijin Hospital, Shanghai Jiaotong University School of Medicine, Shanghai, China; 6 Shanghai Key Laboratory of Signaling and Disease Research, School of Life Science and Technology, Tongji University, Shanghai, China; Macau University of Science and Technology, Macao

## Abstract

Alzheimer’s disease (AD) is a progressive neurodegenerative disease that causes substantial public health care burdens. Intensive efforts have been made to find effective and safe disease-modifying treatment and symptomatic intervention alternatives against AD. Smart Soup (SS), a Chinese medicine formula composed of Rhizoma Acori Tatarinowii (AT), Poria cum Radix Pini (PRP) and Radix Polygalae (RP), is a typical prescription against memory deficits. Here, we assessed the efficacy of SS against AD. Oral administration of SS ameliorated the cognitive impairment of AD transgenic mice, with reduced Aβ levels, retarded Aβ amyloidosis and reduced Aβ-induced gliosis and neuronal loss in the brains of AD mice. Consistently, SS treatment reduced amyloid-related locomotor dysfunctions and premature death of AD transgenic *Drosophila*. Mechanistic studies showed that RP reduced Aβ generation, whereas AT and PRP exerted neuroprotective effects against Aβ. Taken together, our study indicates that SS could be effective against AD, providing a practical therapeutic strategy against the disease.

## Introduction

Alzheimer’s disease (AD) is a fatal and progressive neurodegenerative disease which is characterized by the persistent worsening of cognitive function and daily living performance [1], [2], and it has been considered one of the most significant health, social and economic crises of this century. AD is a complex disease, and its pathological hallmarks include senile plaques formed with amyloid peptides, tangles containing twisted tau proteins, destroyed synapses, abnormal brain inflammation, eventual neuronal death and severe brain shrinkage [3]. Current FDA-approved Alzheimer’s drugs are only symptomatic interventions, such as acetylcholinesterase inhibitors and *N*-methyl-D-aspartate (NMDA) receptor antagonists, which are effective only for about half of the patients for approximately 6–12 months [4]. Up till now, no disease-modifying medications have been approved in the last 10 years. Considering that, as with many other complex diseases, the pathogenesis of AD has a multifactorial basis that includes both genetic and environmental risk factors, a successful therapeutic strategy against this disease might be a set of concerted pharmacological interventions that interact with multiple targets including Aβ/tau pathology, abnormal inflammation and neuronal loss.

The formulae of traditional Chinese medicine (TCM) have been well documented in various Chinese ancient literatures as prescriptions for specific ailments. According to the major precepts of TCM, these TCM formulae emphasize not only the symptoms but on restoring and maintaining the body homeostasis, which is very similar to the rationale of modern multi-targeted therapeutics [5], [6]. At present, TCM, as China Food and Drug Administration (CFDA)-approved and controlled medications, is widely practiced side by side with modern western medicine in almost all of China’s hospitals and clinics. TCM herbal formulae, including Smart Soup (SS), which have been applied for many centuries, are still prescribed by Chinese medical physicians to patients with aging-related cognitive impairment. SS, a three-herb formula officially documented in *Gu Jin Yi Jian,* a book published in 1576, is composed of Rhizoma Acori Tatarinowii (AT), Poria cum Radix Pini (PRP) and Radix Polygalae (RP). Each component is frequently used in different TCM formulae for their pharmacological efficacies against dysfunctions of the central nervous system (CNS, Table S1). AT has been shown to exhibit a neuroprotective action and attenuates learning and memory deficits [7]. PRP has been reported to possess sedative activity [8]. RP shows repairing effects on the memory and behavioral deficits in rats [9], exhibits neuroprotective effects [10], [11], enhances cognition and memory in elderly adults [12], [13]. This study was to assess the efficacy of SS against AD.

## Materials and Methods

### Ethics Statement

All animal experiments were performed according to the National Institutes of Health Guide for the Care and Use of Laboratory Animals. The animal protocols were approved by the Biological Research Ethics Committee, Shanghai Institutes for biological Sciences, Chinese Academy of Sciences. Animal pain and discomfort were minimized with efforts.

### Preparation and the quality analysis of SS

The drug materials were purchased and identified according to the rigid specifications set by *Chinese Pharmacopeia* (2010 Edition). The CFDA-approved single-herb granules of Rhizoma Acori Tatarinowii (AT), Poria cum Radix Pini (PRP) and Radix Polygalae (RP) were obtained from Tianjiang Pharmaceutical, Jiangyin, China. The granule-mixed Smart Soup (SS-G) were prepared by mixing 10 g of AT, 10 g of PRP and 10 g of RP granules to a concentration of 1 g/ml in water.

The chemical constituent identification of each batch of SS was performed using HPLC-TOF/MS. In detail, an aliquot of 1 ml of SS-G was centrifuged at 12,000 rpm. The supernatant was filtered and used for analysis. HPLC-TOF/MS was performed on a 1200 Series HPLC instrument (Agilent, Waldbronn, Germany) coupled with an Agilent 6224 Accurate-Mass TOF LC/MS. The chromatographic separations were performed at 25°C on an Apollo-C18 reversed-phase column (4.6×250 mm i.d., 5 µm, Grace) connected to an EasyGuard Kit C18 guard column (4×2 mm, Grace). The separation was conducted with an acetonitrile/water gradient with 0.5% formic acid. The injection volume was 20 µl for MS analyses.

### Quality analysis of SS using HPLC fingerprints

To assure the quality and thereby warrant the safety and effectiveness of the SS, the chromatographic fingerprints of SS were established and characterized using HPLC. Detection was performed at a wavelength of 320 nm at room temperature. Similarity analysis was performed using similarity evaluation system for TCM chromatographic fingerprints (Version 2004A, Chinese Pharmacopeia Commission) as recommended by CFDA.

### APP/PS1 transgenic mice and drug treatment

The APPswe/PS1dE9 (APP/PS1) double-transgenic mice (The Jackson Laboratory, stock number 004462) were used in our investigation [14]–[16]. The mice were maintained and genotyped according to the guidance of Jackson Laboratory. The transgene-negative wild type (WT) littermates were used as age-matched controls.

APP/PS1 and WT mice were chronically administered 200 µl of SS (1 g/ml) or vehicle only (water) per 20 g mouse body weight by gavage once per day from 7 to 9 months old (n = 8–12 mice per group).

### Morris water maze test

The Morris water maze (MWM) was performed as described [17]–[19]. The apparatus was a circular pool of 120 cm diameter filled with water with small white plastic balls maintained at 23.0±0.5°C. A transparent platform of 11 cm diameter 1 cm below the water surface was placed at a fixed point of one quadrant. Animals were brought to the behavior room, acclimatized and trained.

The training consisted of 10 consecutive days, with four trials per day. On day 4 and 7, a probe trial was performed, followed by four training trials. On the 11th day, a single probe trial was conducted. Swim paths were monitored using an automated tracking system (Ethovision XT software).

### Objective recognition test

Tests were performed as previously described [20], [21] with modifications. The detailed procedures are schematically represented in Fig. S1. The apparatus consisted of an evenly illuminated soundproof box with a Plexiglas box (25 cm×25 cm×25 cm) inside. The procedure included four phases: pre-habituation, habituation, training and testing. The animals were familiarized with the environment for at least one day. On the 1st day of the experiment, the mice were randomly ordered and habituated to the empty box for 5 min. On the 2nd and 3rd day, each mouse was allowed to freely explore two identical objects, which were located at points with same distance from the nearest corner. On the 4th day, during the training phase, each mouse was allowed to explore the identical objects for 10 min first. After a one-hour interval, during the 10-min testing phase, the mouse was returned to the same box with one familiar object switched to a novel one.

To preclude the existence of olfactory cues, each mouse had its own packing paper in the box that wiped thoroughly before each testing.

Object exploration time was the length of time when a mouse was sniffing, directing its nose to and pawing the object. The exploration time was recorded in a double-blinded manner. The location preference in the training phase and recognition index in the testing phase were calculated as following: Location preference means the time exploring one object relative to the time exploring two objects, and Recognition index means the time exploring the novel object relative to the time exploring two objects.

### Immunohistochemistry and image analysis

The mice were anesthetized and transcardiac perfused with phosphate-buffered saline (PBS) buffer and then with 4% paraformaldehyde (PFA) in PBS. 30-µm thick brain sections were prepared and immunostained using a mouse antibody against Aβ (6E10, Covance) for amyloid plaques, a polyclonal rabbit antibody against GFAP (DAKO) for astrocytes, a mouse antibody against CD11b (BD) to assess microglia or anti-NeuN antibodies (Millipore) to assess neurons. Images were captured using a microscope (Carl Zeiss). Quantification was performed using Image-Pro Plus 5.1 software (Media Cybernetic), and the percentage of antibody-immunoreactive area was calculated. The plaques were identified as area >1,500 µm^2^, and the number of plaques were also calculated as three groups: 1,500 µm^2^< plaque size <3,000 µm^2^; 3,000 µm^2^<plaque size <6,000 µm^2^; plaque size >6,000 µm^2^. Four to five coronal sections were analyzed per mouse.

### ELISA for Aβ

Hippocampal and cortical extracts were prepared as previously reported [22]. The accumulation of human Aβ_40_ and Aβ_42_ in these extracts was quantified using ELISA kits (ExCell Bio). Fly head extracts were prepared and the Aβ levels were assessed as reported [23]. Aβ_40_ and Aβ_42_ in SK-N-SH-APPsw cell or HEK293-APPsw cell culture medium were also measured with ELISA.

### Drosophila culture and drug treatments

We used the pan-neuronal elav-GAL4 to express transgenes as described [24]. The upstream activating sequence (UAS) transgenic lines of Aβ_42_ and APP/BACE were provided by Dr. FD Huang and have been described in detail [25]. Canton S (CS) flies were used as WT controls. These flies were kept in a 25°C incubator with 65% humidity and 12 h light/12 h dark circle. The drugs were mixed with liquid food, 0.5 g of instant food (Q/SCQC0005S, Nestle) and 0.05% methyl p-hydroxybenzoate (Sinopharm) in a total of 1.4 ml of sterile distilled water. Every 3 days, the fly food was changed.

### Survival assay

Sixty flies of each group were cultured at 25°C. The number of dead flies was recorded every day. Survival rates were calculated using the Kaplan–Meier estimation.

### Locomotor assay

The locomotor assay of flies was performed as described previously with minor modifications [26]. Briefly, treated ten male flies (n = 30 for each group) were placed in a plastic 25-ml tube. After a 30-min recovering phase, flies were gently tapped to the bottom of the tube. The fly behavior was recorded with a video camera. After 10 s of climbing, the number of flies between the 0, 5, 10, 15, 20 and 25 ml scale marks were recorded (Fig. S2). The results for each group of flies are calculated by the formula below:



Climbing Index  =  (flies above 20 ml scale mark) × 1 + (flies between 15 and 20 ml scale marks) × 0.8 + (flies between 10 and 15 ml scale marks) × 0.6 + (flies between 5 and 10 ml scale marks) × 0.4 + (flies below 5 ml scale mark) × 0.2.

### Western blot analyses

Fly head lysate preparation and western blot analyses were performed as described previously [23], [27]. Briefly, thirty fly heads from each group were collected and lysed in RIPA buffer containing protease inhibitors. HEK293-APPsw cell lysates were also prepared. Proteins were separated on 16% tricine gel and probed with antibodies against APP C-terminal and β-actin (Sigma-Aldrich).

### Preparation of Aβ peptides

The peptides were prepared according to the protocols described by Stine [28]. Briefly, hexafluoroisopropanol (HFIP)-treated Aβ_42_ peptides (Anaspec) were resuspended in dimethyl sulfoxide (DMSO). For the oligomeric conditions, the peptide was then diluted to a concentration of 100 µM with Ham’s F12 and incubated at 4°C for 24 hours. After centrifugation 10 min at 14,000 g, the supernatant with soluble Aβ_42_ oligomers was added to cultures. The soluble oligomeric Aβ peptides were confirmed using atomic force microscopy (AFM) and western blot analysis.

### Thioflavin-T fluorescence assay

The Thioflavin-T (Th-T, Sigma-Aldrich) fluorescence assay was performed to measure amyloid fibril formation [29]. Th-T was prepared as a stock at 2 mM (avoid exposure to light) and filtered through a 0.22 µm filter. The Th-T stock solution was diluted into PBS on the day of analysis. HFIP-treated Aβ peptides were dissolved in DMSO at 5 mM, and then diluted in Th-T/PBS with a final concentration of 15 µM (Aβ) and 20 µM (Th-T). The Aβ/Th-T solutions were incubated at 37°C with or without TCM for six days in a black 96-well-plate (PerkinElmer). Fluorescence was measured using an EnVision multilabel plate reader (PerkinElmer), at excitation and emission wavelengths of 440 nm and 490 nm, respectively. Fluorescence of 20 µM Th-T/PBS solution was measured and used as blank. The fluorescence intensity reflects the degree of Aβ aggregation.

### Primary culture of neuron

Primary neurons were cultured as described previously [30], [31]. The cortices and hippocampi obtained from newborn C57BL/6 mice were used. 5×10^4^ cells per well in DMEM/Ham’s F12 containing 10% FBS (Invitrogen) were plated onto poly-D-lysine (Sigma-Aldrich) coated 96-well plates and maintained at 37°C for four hours, and the culture medium was then switched to B27/Neurobasal medium (Invitrogen). On day 6, the cells pre-treated with TCMs for two hours were incubated with 5 µM Aβ_42_ oligomers and TCMs for another 48 hours. Staining of primary neurons with a Tuj1 antibody (Covance) was imaged and analyzed using Operetta (PerkinElmer).

### CellTiter-Glo assay

The primary neurons, SK-N-SH-APPsw cells and HEK293-APPsw cells were assessed for viability using the CellTiter-Glo luminescent cell viability assay (Promega) following the manufacturer’s instructions.

### TUNEL assay

The assay was performed using the Kit from Roche following the manufacturer’s protocol. Primary neurons were pre-treated with TCMs for two hours followed by incubation with 5 µM Aβ_42_ oligomers and TCMs for another 24 hours. The total numbers of DAPI-stained or TUNEL-positive cells were counted.

### Atomic Force Microscope (AFM)

Amyloid peptides for AFM analysis were prepared according to the protocols described by Stine [28]. First, HFIP-treated Aβ_42_ peptides (Anaspec) were dissolved in DMSO. 5 mM Aβ_42_ in DMSO were diluted in H_2_O to 100 µM and used immediately as Aβ_42_ monomers. 5 mM Aβ_42_ in DMSO were diluted in Ham’s F12 to 100 µM and incubated at 4°C for 24 hours and used as Aβ_42_ oligomers. Aβ_42_ fibrils were prepared by diluting 5 mM Aβ_42_ in DMSO with 10 mM HCl to 100 µM and incubated at 37°C for 24 hours. For AFM analysis, Aβ_42_ monomers, oligomers and fibrils samples were further diluted to a final concentration of 10 µM with distilled H_2_O and spotted on freshly cleaved mica disks for 3 minutes. After being rinsed three times with deionized water, the mica disks were dried overnight at room temperature. AFM was performed in tapping mode using a Molecular Force Prove 3D (MFP-3D, Asylum Research) with NSC11 cantilevers (MikroMasch, 48 Newton/meter spring constant). The data were analyzed with Igor-Pro (Wavemetrix). The resonance frequency was applied in the 330 kHz range, the scan rate was 1 Hz, and the resolution was 512×512 pixels.

### Statistical analyses

GraphPad Prism 5.0 was used. The data are shown as the mean ± S.E.M. The Kaplan-Meier test was employed to compare the differences between the survival curves using SPSS 16.0. Group differences were analyzed with one-way ordinary or repeated-measures analysis of variance (ANOVA) followed by Tukey multiple comparisons test. Student’s independent *t* test was applied for comparisons of two groups. Differences were significant when *P*<0.05.

## Results

### SS treatment ameliorates learning and memory deficits in APP/PS1 mice

Traditionally, TCM formulae are prepared as decoctions, i.e., the herbs are boiled for a fixed time, and the liquid is used. Recently, CFDA-approved single-herb granules have been widely accepted as alternatives for decoctions. These CFDA-approved single-herb granules are clearly documented for their chemical fingerprints and pharmacokinetic parameters along with standard manufacturing protocols, which are not only suitable for large-scale industry production but are also more appropriate for evaluation of their efficacies and exploration of the underlying molecular mechanisms. Thus, as described in the Methods section, we prepared SS using the CFDA-approved single-herb granules according to the recipe (granule-mixed SS, SS-G). We first analyzed the SS-G by HPLC-TOF/MS. The total ion current chromatograms corresponding to positive and negative signals of SS were obtained, as shown in Fig. S3 and Table S2. We found that the major chemical constituents identified in the SS-G were similar to the traditionally prepared SS decoctions (SS-D) [32]. Therefore, we used the SS-G for the following assays. Then, we compared the HPLC fingerprints of four different batches of SS-G. The similarity indices of these four batches of SS were between 0.943 and 0.982 (Fig. S4), demonstrating that SS was produced consistently with good quality.

**Figure 1:**
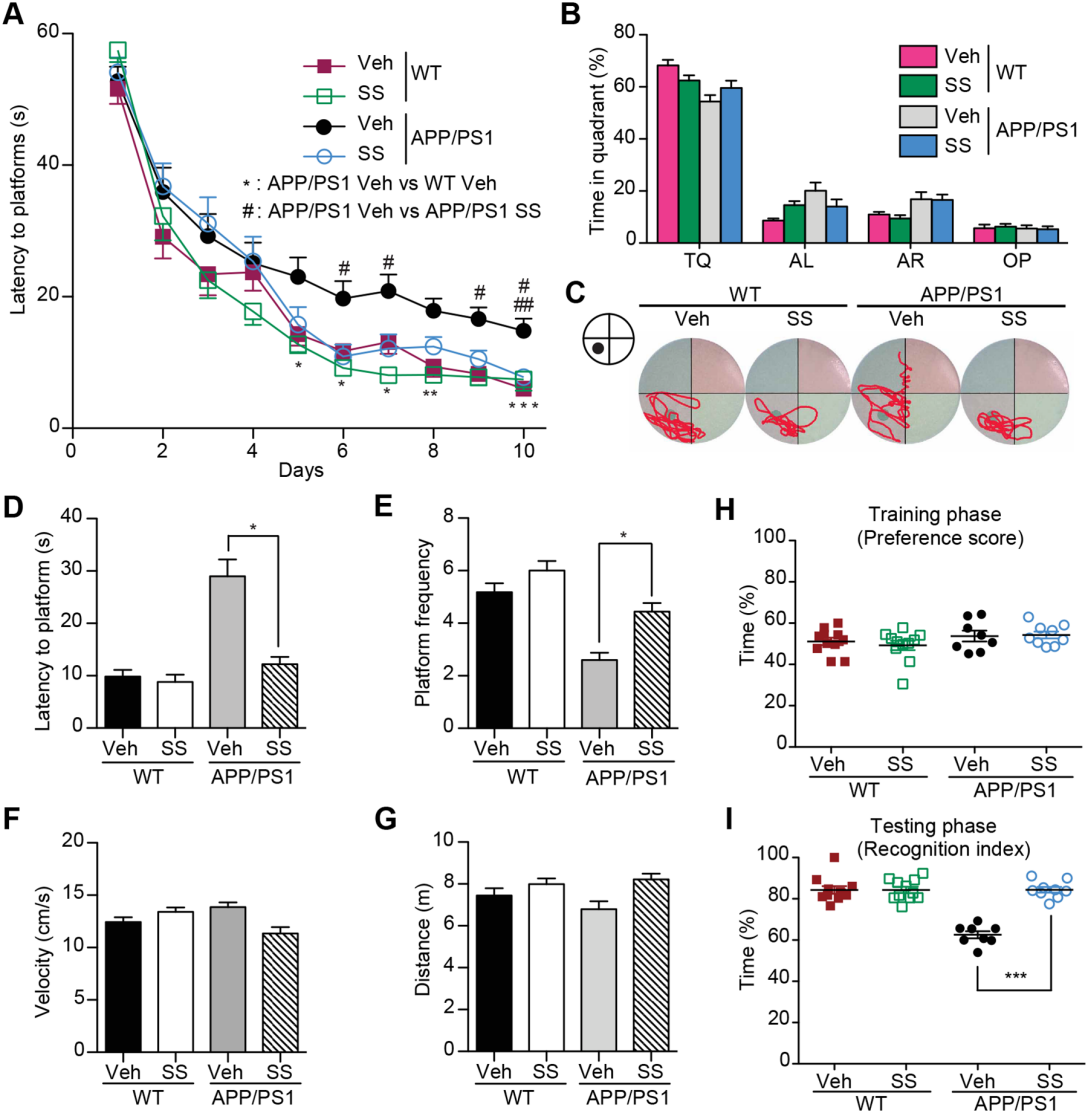
SS treatment ameliorates learning and memory impairment in Morris Water Maze and Object recognition test. (**A**) MWM test for SS and vehicle-treated APP/PS1 and WT mice. The mean escape latency was given for different test days. (**B**) The mean percent time in probe trial of MWM on day 7. TQ: Target quadrant; AL: Adjacent left; AR: Adjacent right; OP: Opposite. (**C**) Representative mice search paths from different groups. (**D and E**) The latency to target quadrant (**D**) and the frequency to pass the target position (**E**) in probe trial are shown. (**F and G**) The swimming velocity (**F**) and distance (**G**) in probe trial are shown. (**H and I**) Novel object recognition analysis. Preference scores of training phase (**H**) and Recognition Index of testing phase (**I**) during a 10-min testing phase are shown, respectively. n = 9–12 for each group. *P<0.05, **P<0.01, ***P<0.001, #*P*<0.05, ##*P*<0.01, ###*P*<0.001.

The age-dependent Aβ accumulation was found in the brains of APP/PS1 mice, and memory deficits were began to show since the age of 6 months [33]. To explore the potential therapeutic effects of SS, we began the oral administration of SS to the 7-month-old APP/PS1 mice for two months. There were no obvious adverse effects or body weight loss (Table S3). We compared the mice behavioral phenotypes to assess the cognitive function of these transgenic AD mice after drug administration. We assessed the spatial memory of these mice in the MWM. The MWM is a common behavioral task used to determine spatial learning and reference memory deficits. SS treatment did not influence the swimming velocity and distance (Fig. 1, F and G). However, the APP/PS1 mice showed defects in learning compared with the WT mice (Fig. 1A). On day 5, vehicle-treated APP/PS1 mice spent more time than WT littermates to locate the hidden platform (*P*<0.05, APP/PS1 mice *vs.* WT mice, Fig. 1A). This difference constantly appeared on day 6, 7, 8 and 10, reflecting deficits in spatial memory of APP/PS1 mice, as reported previously [34]. Interestingly, since day 6, SS-treated APP/PS1 mice showed an improved performance compared with vehicle-treated APP/PS1 mice (*P*<0.01, Fig. 1A), indicating that the SS treatment alleviated the impairment of spatial learning in APP/PS1 mice. To assess the memory strength of spatial learning, we administered the probe trials on day 4, 7 and 11. The mice in all group showed a random swimming pattern on day 4 (Fig. S5). However, on the day 7 probe trial, compared with the vehicle-treated APP/PS1 mice, the SS-treated group spent more time searching for the platform in the target quadrant (Fig. 1, B and C), took less time to reach the position of the missing platform (*P*<0.05, Fig. 1D), and increased frequency of crossing within the position of the platform (*P*<0.05, Fig. 1E). These results suggest that administration of SS alleviates the deficient spatial reference memory of APP/PS1 mice.

To further assess the learning and recognition memory processes of these AD mice, we applied the object recognition test (Fig. S1). There were no obvious differences among mice in all groups regarding which object they preferred or the location of the objects as the preference scores were all approximately 50% (Fig. 1H). In the testing phase, the WT mice significantly increased the time exploring the novel object (*P*<0.001, Fig. 1I). In contrast, the time exploring the novel object of vehicle-treated APP/PS1 mice did not increased obviously. Meanwhile, the SS-treated APP/PS1 mice explored the novel object longer than vehicle-treated APP/PS1 mice (*P*<0.001) indicating improved memory retention by SS treatment.

### SS treatment reduced Aβ levels, Aβ amyloidosis, gliosis and neuron loss in the brains of APP/PS1 mice

**Figure 2:**
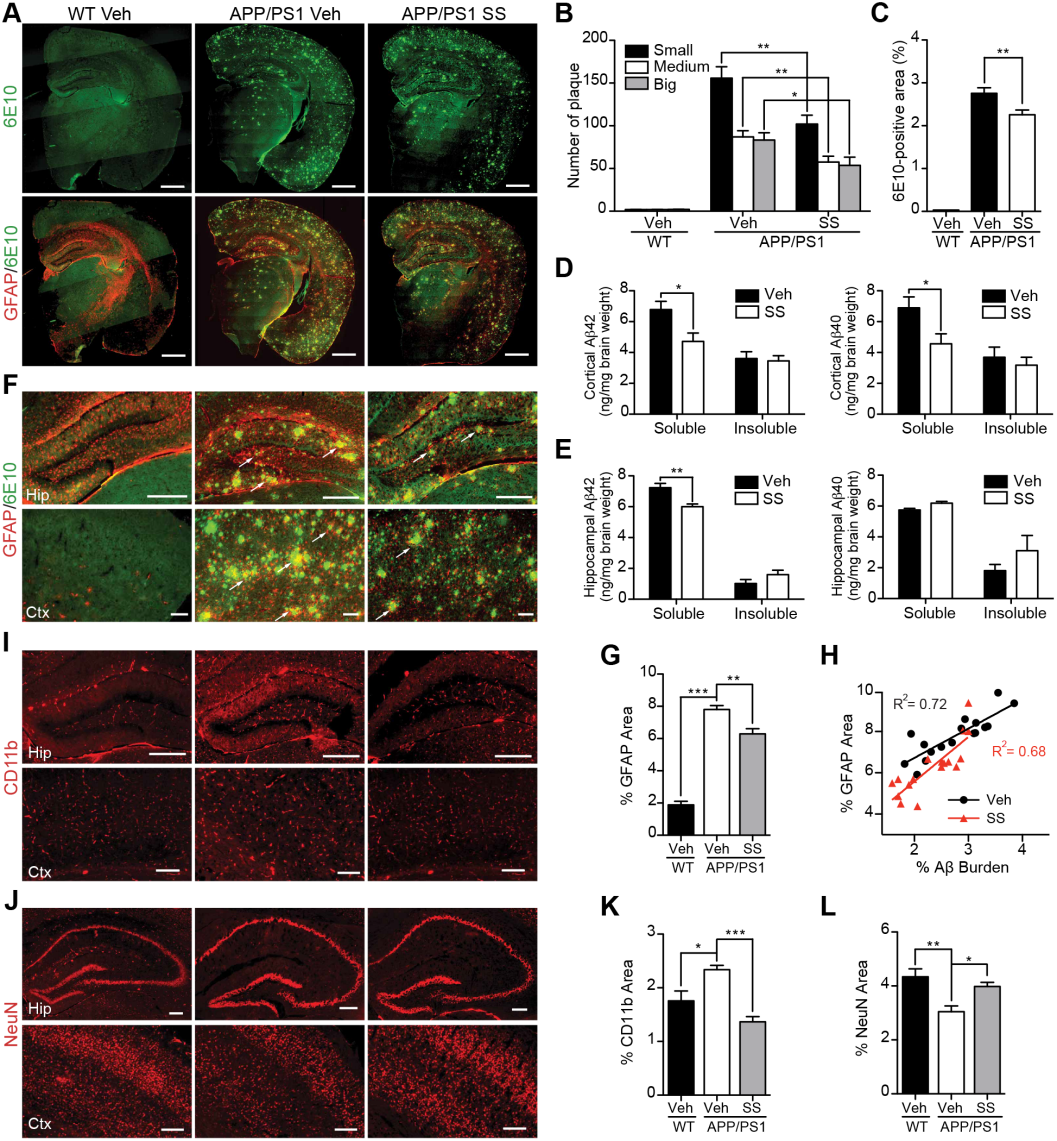
SS treatment alleviates Aβ levels and amyloid plaque burden, reduces gliosis and neuron loss in APP/PS1 mice. (**A–C**) Representative half brain sections of WT mice, vehicle or SS-treated APP/PS1 mice stained with antibody against Aβ (6E10) and double staining of GFAP and 6E10 are shown. Scale bar, 1 mm. (**B** and **C**) Quantitative analysis of the number of 6E10-positive amyloid plaques (**B**) and Aβ covered area (**C**). n = 5 animals per group. (**D** and **E**) ELISA of soluble and insoluble Aβ_40_ and Aβ_42_ levels in cortical and hippocampal tissues of APP/PS1 mice. n = 6 for each group. (**F**, **I** and **J**) Representative images of WT mice, vehicle- and SS- treated APP/PS1 mice hippocampus and cortex double immunostaining of GFAP and 6E10 (**F**), CD11b (**I**) and NeuN (**J**). Arrows indicate astrocytes surrounding the amyloid plaques. Scale bar, 200 µm. (**H**) Coincidence of GFAP and Aβ burden in the brains of SS-treated APP/PS1 mice (red; n = 17) and vehicle-treated APP/PS1 mice (black; n = 17; *P*<0.0001). (**G**, **K** and **L**) The histograms depict the mean GFAP (**G**), CD11b (**K**), and NeuN (**L**) positive area ± S.E.M. in three groups. *P<0.05, **P<0.01, ***P<0.001.

APP/PS1 mice begin to develop cerebral amyloidosis at 2 months of age, and the Aβ levels or Aβ deposits in the brain can be detected since 6 months of age. Histology was performed on fixed brain tissues. APP/PS1 mice showed 6E10-positive amyloid plaques (Fig. 2A), which were significantly reduced in the brains of SS-treated APP/PS1 mice. Detailed plaque size distribution analysis revealed a reduction of different size plaques in SS-treated APP/PS1 mice compared with that of control APP/PS1 mice (Fig. 2B). The overall amyloid plaques of SS-treated APP/PS1 mice was reduced by 18.1% compared with that of control APP/PS1 mice (*P*<0.01, Fig. 2C), indicating that SS treatment prevented the formation of plaques and/or enhanced the clearance of amyloid plaques.

Soluble Aβ oligomers are deleterious, and related to cognitive deficits in AD [35]–[40]. To quantify Aβ levels, we used an ELISA assay. ELISA analysis of SDS-soluble fractions and SDS-insoluble fractions demonstrated that there were high Aβ levels in both fractions from the hippocampi and cortices of APP/PS1 transgenic mice. SS treatment reduced cortical SDS-soluble Aβ_40_ and Aβ_42_ levels (30.4% reduction for Aβ_42_, *P*<0.05, and 33.7% reduction for Aβ_40_, *P*<0.05, Fig. 2D). We also observed a similar reduction of hippocampal SDS-soluble Aβ_42_ level after SS treatment (17.0% reduction, *P*<0.01, Fig. 2E). There were no significant differences between the SDS-insoluble formic acid-extractable fractions from vehicle-treated and SS-treated mice (Fig. 2, D and E) suggesting that SS reduces the level of toxic soluble Aβ. These data indicate that SS treatment reduces Aβ levels and the correlated amyloidosis in APP/PS1 mice.

Abnormal neuroinflammation, including accumulation of activated microglia and astrocytes, is a pathological characteristic of the neurodegenerative disease [41]. In the brains of AD patients and APP/PS1 transgenic mice, amyloid plaques are surrounded by activated microglia and reactive astrocytes [41]. Consistent with previous reports [42], we noted that GFAP-positive astrocytes surrounded the amyloid plaques in APP/PS1 mice (Fig. 2, A and F, arrows), and the GFAP-positive staining intensity in APP/PS1 mice was higher than that of WT mice (7.80±0.25 *vs.* 1.89±0.22 percent area, *P*<0.001, Fig. 2, F and G). Furthermore, coincident GFAP and Aβ staining was obvious in the brains of vehicle-treated APP/PS1 mice (*R^2^* = 0.72, Fig. 2H). CD11b-positive microglia were also observed (Fig. 2I). We found significant reductions of astrocytes (staining intensity: 6.30% *vs.* 7.80%, *P*<0.01; coincidence: 0.68 *vs.* 0.72, *P*<0.001, Fig. 2, G and H) and microglia (staining intensity: 1.37% *vs.* 2.34%, Fig. 2, I and K) in the brains of SS-treated mice, demonstrating that the AD-like pathological gliosis has been significantly moderated by SS treatment in APP/PS1 mice.

As one of the major *in vivo* neurotoxic properties of Aβ, severe neuronal loss is also observed in APP/PS1 transgenic mice since the age of 10 months [43]. Consistently, in addition to the accumulation of microglia and astrocytes, we also observed significant less neurons (NeuN-positive cells) in the brains of APP/PS1 mice compared with WT mice (Fig. 2, J and L). Interestingly, we found an increase in neurons (NeuN-positive cells) in SS-treated APP/PS1 mice compared with control APP/PS1 mice (*P*<0.05, Fig. 2L). Together, these results suggest that in APP/PS1 mice, SS treatment retards the Aβ-related pathological gliosis and neuronal loss.

### SS treatment improves locomotor functions and prolongs lifespan of AD transgenic *Drosophila*

Transgenic *Drosophila* models expressing human Aβ, APP or secretases have been investigated to gain insight into disease mechanisms as well as to elucidate potential therapeutic approaches [44]. AD transgenic *Drosophila* expressing human Aβ_42_ show memory deficiencies and premature death [45]. Very recent reports have also shown that the generation of Aβ oligomers through the APP processing by secretases in APP/BACE flies display a very similar pathology as that in AD patients [46]. Because the AD-like pathologies are evident within a few days in these *Drosophila* models, we performed a rapid *in vivo* comparison of SS *vs.* Memantine, the clinically approved medication for treatment of Alzheimer's disease, using transgenic flies carrying Aβ_42_ or APP/BACE.

We applied the climbing assay to assess the CNS dysfunction of these AD transgenic flies. The climbing assay is a behavioral test based on the negative geotaxis response of *Drosophila*. Thus, assessing the climbing ability of flies in a fixed time period can reflex their locomotor function.

**Figure 3:**
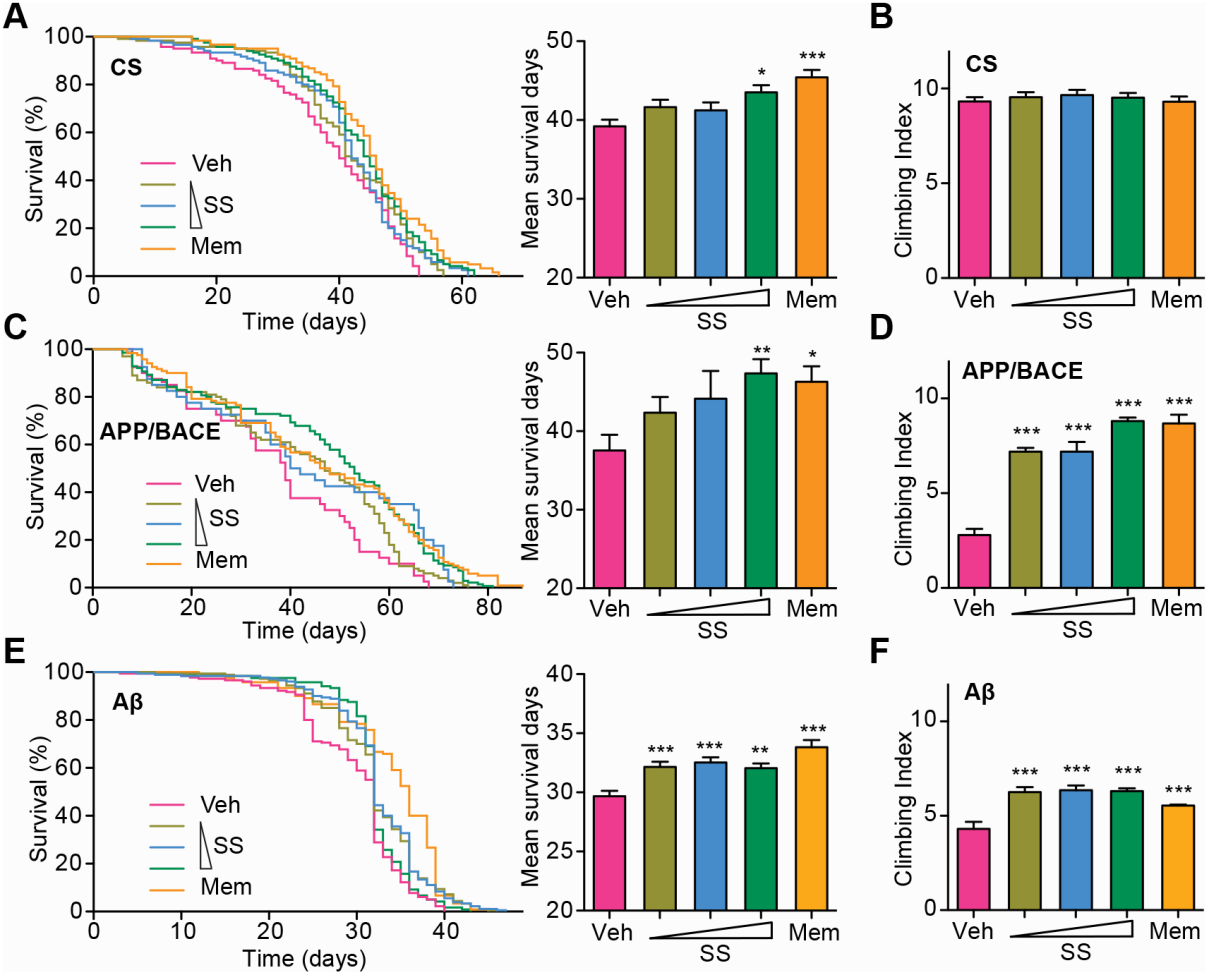
SS treatment improves locomotor functions and prolongs lifespan of AD transgenic *Drosophila*. (**A, C and E**) CS, APP/BACE and Aβ transgenic flies were cultured on food containing different concentrations of SS (the triangle symbol stands for concentrations from low to high: 0.2, 0.6 and 2 mg/ml) or Memantine (120 µM). Survival curves for flies treated with either SS or Memantine. The data are presented as mean ± S.E.M. The right panel shows the mean survival days calculated according to the survival curves. (**B, D and F**) The climbing ability of CS, APP/BACE and Aβ transgenic flies treated with SS or Memantine at day 30 (for CS and APP/BACE flies) and day 20 (for Aβ flies). Values are mean ± S.E.M. Each value represents the mean of three experiments. *P<0.05, **P<0.01, ***P<0.001 *vs.* Ctrl group. Mem = Memantine.

Consistent with previous reports, flies expressing human APP and BACE or flies expressing Aβ_42_ showed premature death compared with CS flies (Fig. 3, A, C and E). We also observed significant decreases in the climbing ability of these flies (Fig. 3, B, D and F). Interestingly, when the Aβ_42_ and APP/BACE transgenic flies were cultured on either SS or Memantine, Aβ-induced premature death was significantly reduced (Fig. 3, C and E). Furthermore, SS or Memantine treatment considerably rescued the climbing ability of these transgenic flies (Fig. 3, D and F). These results indicate that SS treatment improves locomotor functions and reduces premature death of AD transgenic flies, similar to that of Memantine.

### RP reduces Aβ generation

**Figure 4:**
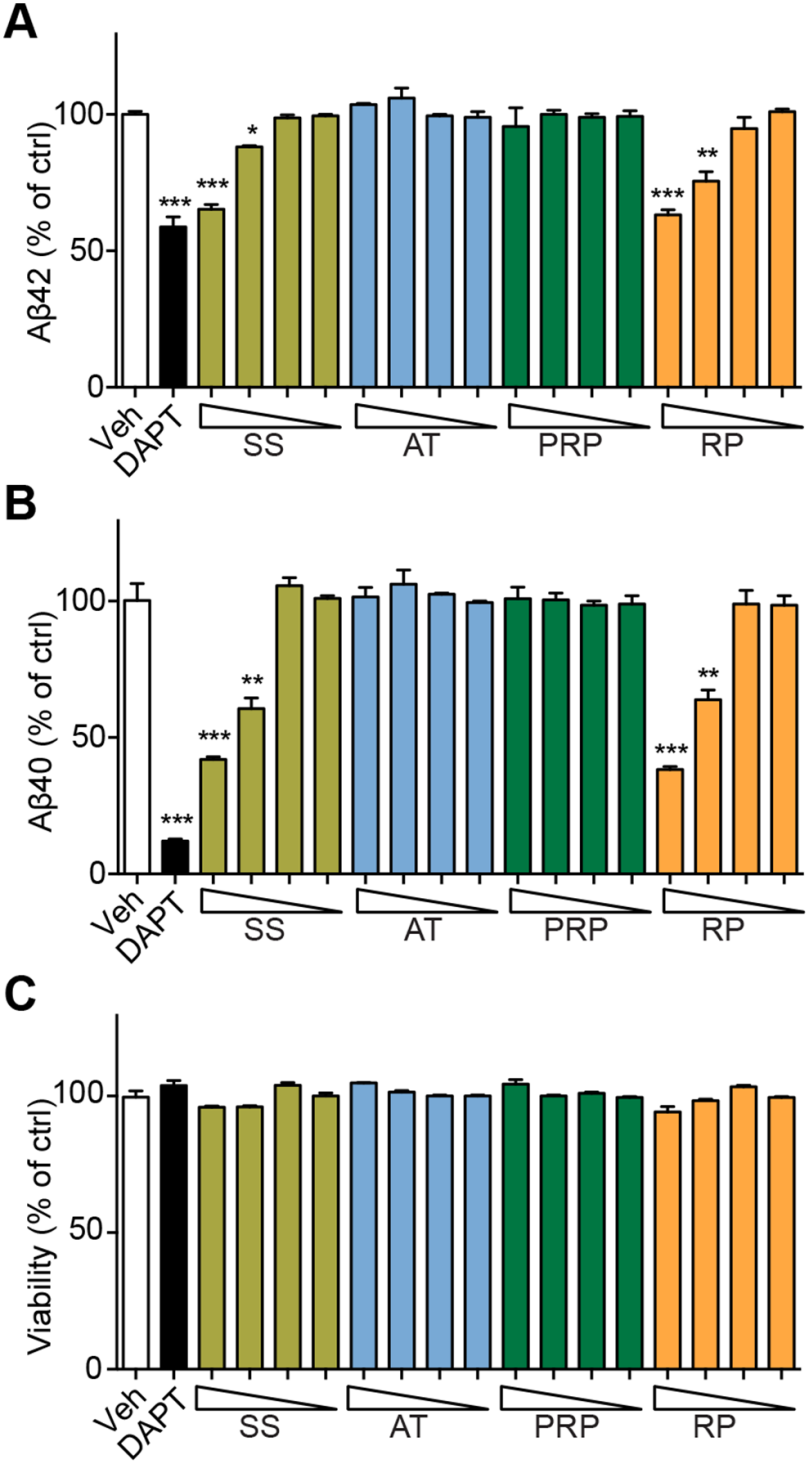
RP reduces the Aβ generation in SK-N-SH-APPsw cells. Aβ_42_ (**A**) and Aβ_40_ (**B**) in SK-N-SH-APPsw cell culture medium and cell viability (**C**) after treatment with SS, AT, PRP, RP for 24 hours, respectively (the triangle symbol stands for concentrations from high to low: 3000, 1000, 300 and 100 µg/ml for SS; 1000, 300, 100 and 30 µg/ml for AT, PRP and RP). *P<0.05, **P<0.01, ***P<0.001; DAPT: a γ-secretase inhibitor.

To explore which granule in SS might reduce Aβ levels, we treated SK-N-SH-APPsw cells with different concentrations of each granule in SS. SK-N-SH-APPsw cells stably express APP protein carrying a Swedish mutant (K595N/M596L) and show elevated Aβ_40_ and Aβ_42 _secretion. As shown in Fig. 4, Aβ_40_ and Aβ_42_ levels in the culture medium of cells treated with SS were reduced in a dose-dependent manner. Interestingly, the same dose of RP but not that of AT or PRP exhibited similar Aβ_40_ and Aβ_42_ reduction effects as SS (Fig. 4, A and B). The viability of cells was not altered by any of these treatments (Fig. 4C). Similar results were observed using HEK293-APPsw cells (Fig. S6).

**Figure 5:**
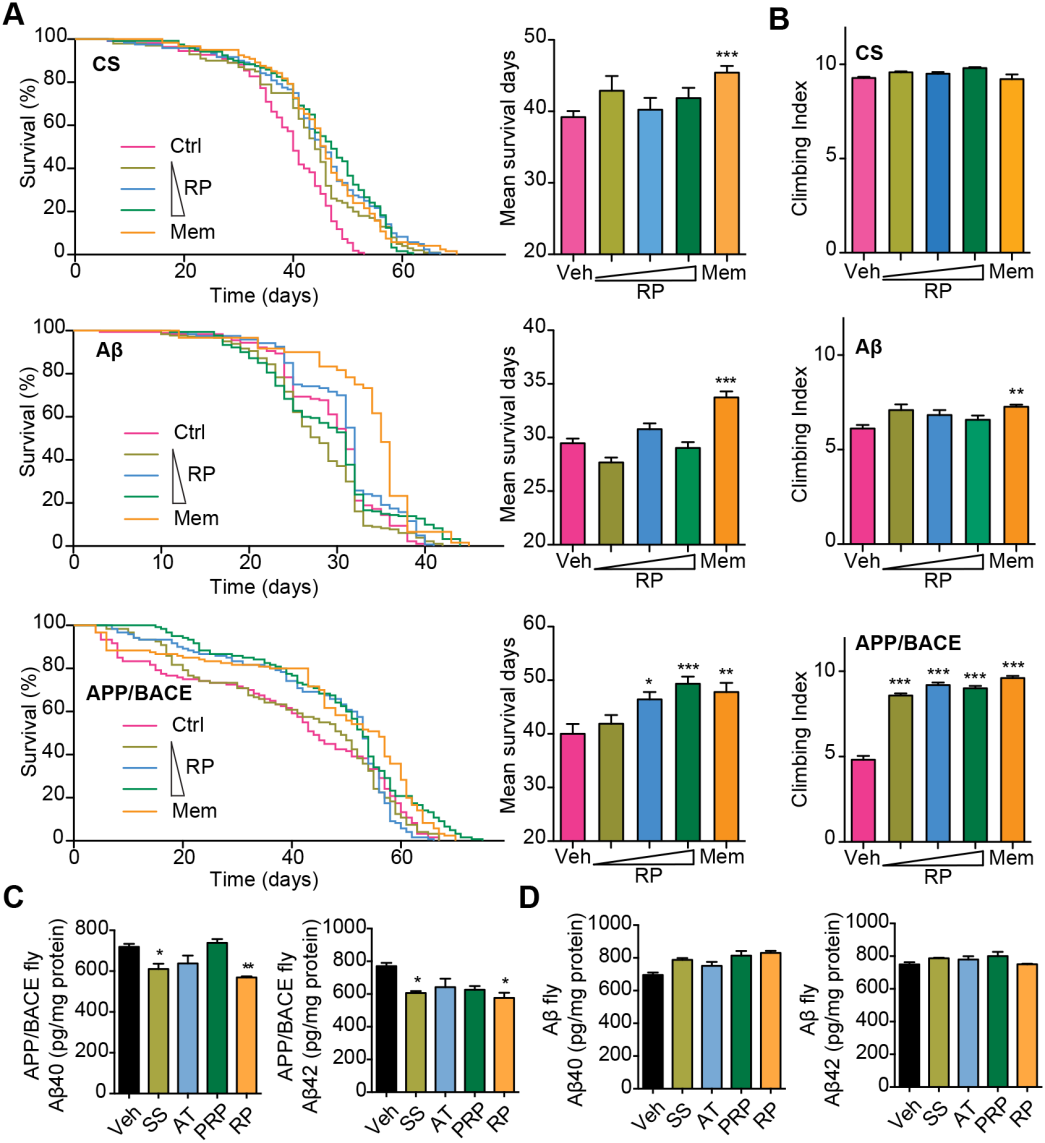
RP treatment improves locomotor functions, prolongs lifespan and reduces Aβ levels of AD transgenic *Drosophila*. CS, Aβ and APP/BACE transgenic flies were cultured on food containing different concentrations of RP (the triangle symbol indicates concentrations from low to high: 0.2, 0.6 and 2 mg/ml) or Memantine (120 µM). (**A**) Survival curves of flies treated with either RP or Memantine. The data are presented as the mean ± S.E.M. (**B**) The climbing ability of flies (right panels) was assessed at day 30 for CS and APP/BACE flies and at day 20 for Aβ flies. The values are the mean ± S.E.M. Each value represents the mean of three experiments. (**C** and **D**) Aβ and APP/BACE transgenic flies were cultured on SS, AT, PRP or RP (2 mg/ml). Aβ_40_ and Aβ_42_ levels in 500 fly heads were measured by ELISA assay. Mem = Memantine. *P<0.05, **P<0.01, ***P<0.001 *vs.* the control group.

Then, we assessed whether RP ameliorates Aβ-related pathology by reducing Aβ generation *in vivo*. We found that RP showed no obvious beneficial effects in the Aβ transgenic flies expressing Aβ_42_ directly (Fig. 5A). Interestingly, APP/BACE transgenic flies that were cultured on food containing RP showed considerably reduced Aβ-induced premature death (Fig. 5B). Consistently, the climbing ability of these RP-treated APP/BACE transgenic flies was enhanced than that of vehicle-treated APP/BACE transgenic flies. Aβ levels in APP/BACE and Aβ transgenic flies were monitored by ELISA. As shown in Fig. 5C, APP/BACE flies cultured on food containing SS or RP produced significantly lower levels of Aβ_40_ and Aβ_42_ than control. Moreover, Aβ levels in Aβ transgenic flies cultured on food containing SS or RP were not different from that of control Aβ transgenic flies (Fig. 5D). This result suggests that RP may affect human APP proteolysis. To further assess the proteolysis process of APP in APP/BACE flies, full-length APP and APP C-terminal fragments (APP-CTF) in fly heads were monitored using western blots. There was no significant difference in full-length APP and APP-CTF levels in APP/BACE flies (Fig. S7). These results suggest that RP can reduce Aβ generation and thus moderate Aβ-related pathology *in vivo*.

### AT and PRP moderate Aβ-induced neurotoxicity *in vivo* and *in vitro*

**Figure 6:**
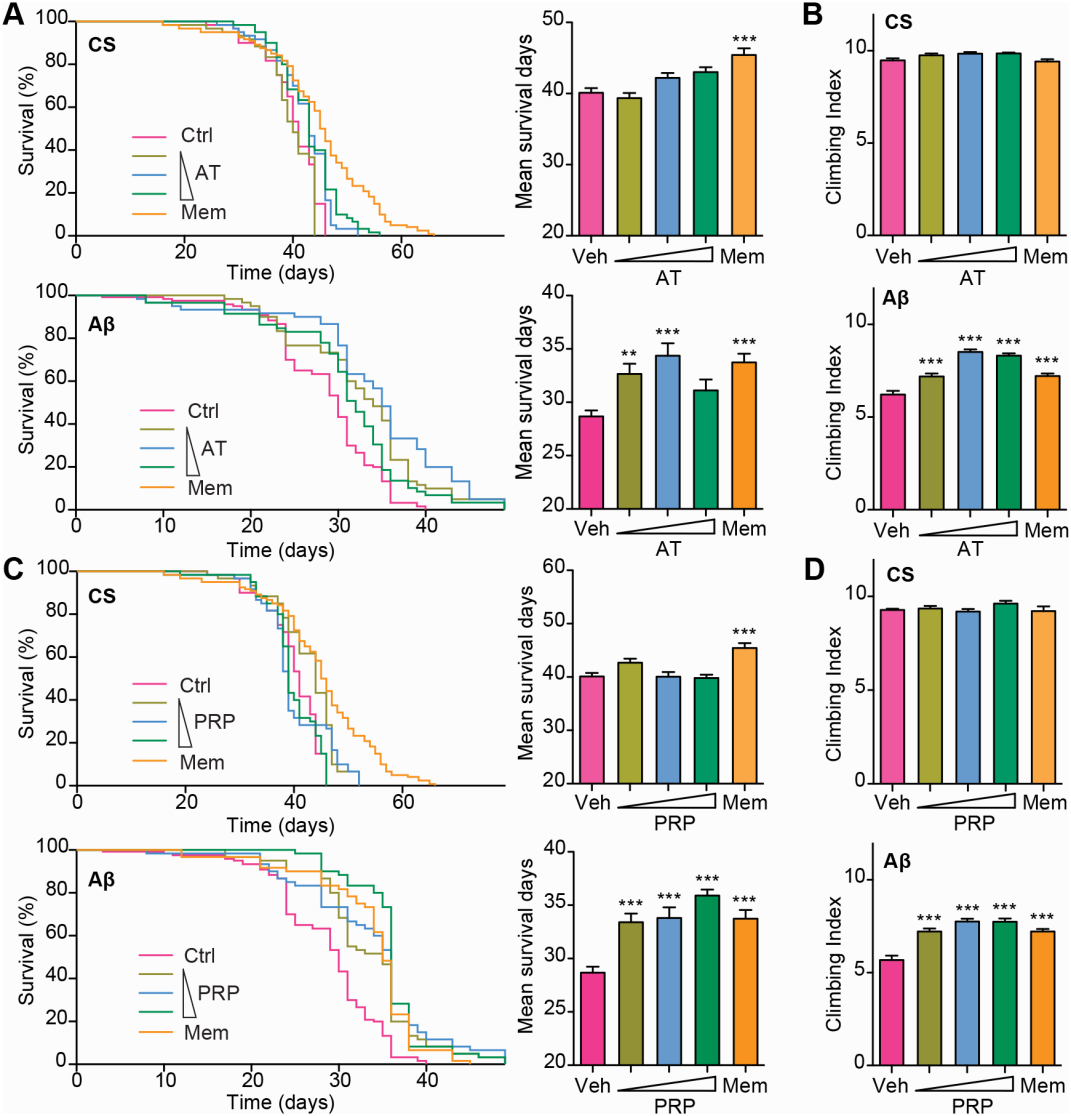
AT and PRP improves locomotor function and prolongs lifespan of AD transgenic *Drosophila*. CS and Aβ transgenic flies were cultured on food containing different concentrations of AT (0.2, 0.6 and 2 mg/ml), PRP (0.2, 0.6 and 2 mg/ml) or Memantine (120 µM). (**A, C**) Survival curves for flies treated with either AT, PRP or Memantine. (**B, D**) The climbing ability of flies was assessed. The values are the mean ± S.E.M. Each value represents the mean of three experiments. Mem = Memantine. *P<0.05, **P<0.01, ***P<0.001 *vs.* the control group.

Aβ-induced neuronal apoptosis in brain is a typical feature of Alzheimer's disease [47]. Transgenic flies carrying the toxic human Aβ_42_ show neuronal dysfunction and premature death and thereby provide a unique system to evaluate potential protective effects against Aβ-induced neurotoxicity *in vivo*. We found that SS but not RP treatment improved locomotor functions and reduced premature death of Aβ transgenic flies (Fig. 3, E and F and Fig. 5, A and B), suggesting that either AT or PRP has a neuroprotective property. We treated Aβ_42_ transgenic flies with different concentrations of AT or PRP. As shown in Fig. 6, both AT and PRP prolonged lifespan and improved the climbing activity of Aβ_42_ flies (Fig. 6, A–D). Both AT and PRP also prolonged lifespan and improved climbing activity of APP/BACE flies but to a less significant extent (Fig. S8).

**Figure 7:**
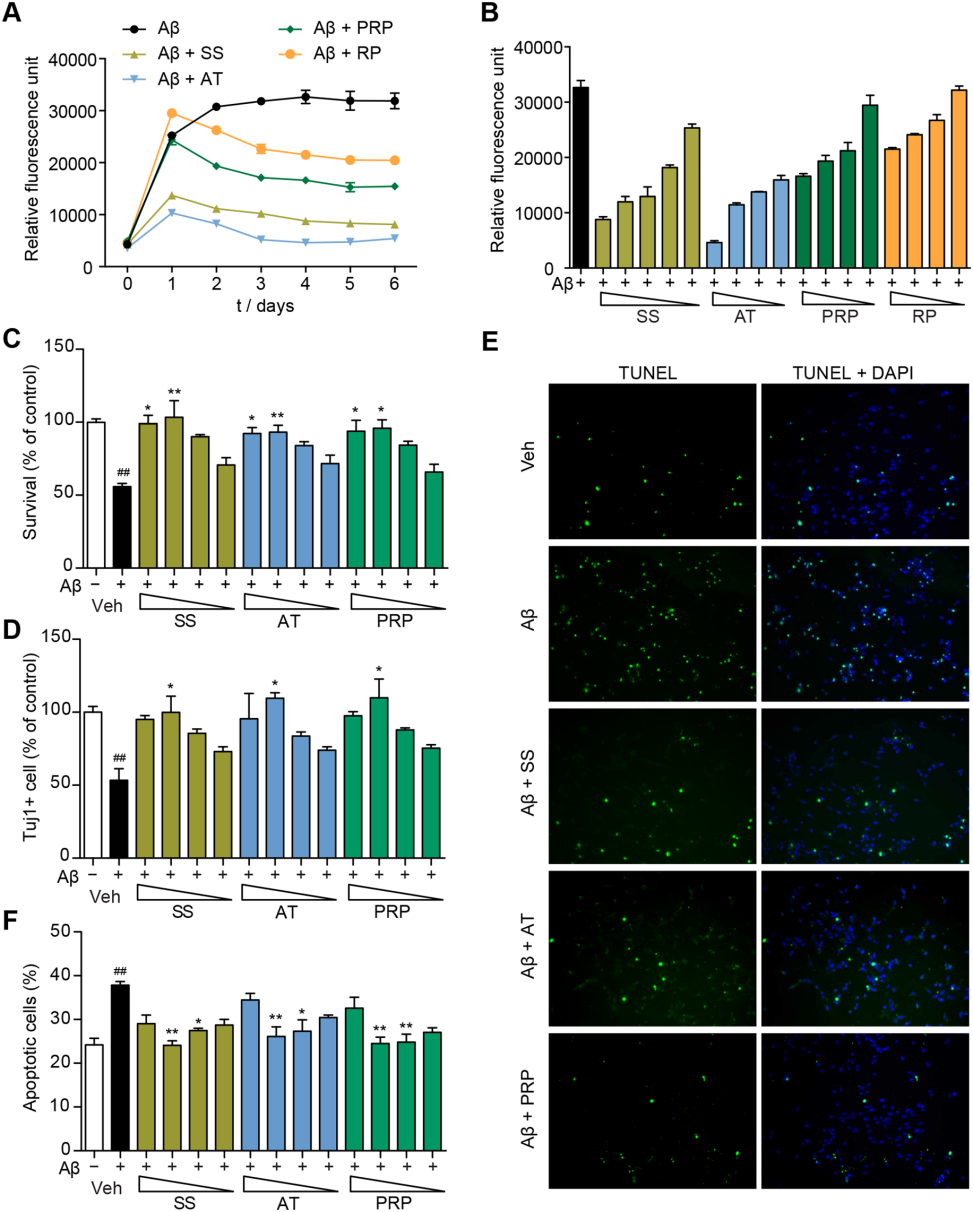
AT and PRP inhibit Aβ_42_ aggregation and exert neuroprotective effects against Aβ_42_ in primary neurons. (**A**) The effects of SS (3000 µg/ml), AT (1000 µg/ml), PRP (1000 µg/ml) or RP (1000 µg/ml) on Aβ_42_ aggregation, as measured by Th-T fluorescence assay. (**B**) SS, AT, PRP and RP (3000, 1000, 300, 100 and 30 µg/ml for SS; 1000, 300, 100 and 30 µg/ml for AT, PRP and RP) inhibited Aβ_42_ aggregation in dose-dependent manner. (**C** and **D**) Cell viability of primary cultured neurons pre-treated with SS or AT, PRP for two hours followed by incubation with Aβ_42_ oligomers (5 µM) for another 48 hours (300, 100, 30 and 10 µg/ml for SS; 100, 30, 10 and 3 µg/ml for AT and PRP). Viable cells were quantified using a CellTiter-Glo assay (**A**). Tuj1-positive cells were counted and presented (**D**). *P<0.05 compared with the Aβ_42_-treated group. (**E** and **F**) TUNEL analysis of the primary neurons pre-treated with SS (100 µg/ml), AT (30 µg/ml), or PRP (30 µg/ml) for two hours followed by incubation with Aβ_42_ oligomers (5 µM) for another 24 hours. The green, TUNEL-positive cells are merged with blue DAPI-positive cells (**E**). The TUNEL-positive cells and DAPI-positive cells pre-treated with SS or AT, PRP (300, 100, 30 and 10 µg/ml for SS; 100, 30, 10 and 3 µg/ml for AT and PRP) were counted in three independent experiments (**F**). *P<0.05, **P<0.01, ***P<0.001 *vs.* the Aβ_42_-treated group, ##*P*<0.01 *vs.* the control group.

To investigate whether SS inhibits Aβ aggregation, we performed a Th-T fluorescence assay. Th-T fluorescence signals were gradual increased over time. Both SS and AT inhibited the fluorescence intensity almost completely (Fig. 7A), while PRP showed a less significant effect. SS, AT and PRP inhibited Aβ_42_ aggregation in a dose-dependent manner (Fig. 7B). To directly assess the neuroprotective effects of AT and PRP, we further monitored Aβ-induced cell death of primary mouse cortical neurons. Aβ_42_ oligomer preparations consistently provided small oligomers and expected structure as monitored by western blot and AFM (Fig. S9), respectively. Aβ_42_ oligomers effectively induced the death of cortical neurons within 48 hours as measured by the CellTiter-Glo assay (Fig. 7C) and Tuj1 staining (Fig. 7D). SS significantly prevented neurons from Aβ_42_ oligomers-induced cell death. Consistent with the *in vivo* results above, both AT and PRP improved primary neuron survival in the presence of toxic Aβ_42_ oligomers (Fig. 7, C and D). Aβ_42_ oligomers-induced cell apoptosis was further monitored using a TUNEL assay. As shown in Fig. 7E, approximately 40% of cells showed severe DNA fragmentation upon Aβ_42_ treatment. In the presence of SS, AT or PRP, the number of apoptotic cells was significantly reduced (Fig. 7, E and F). Together, these data indicate that both AT and PRP protect neurons from Aβ_42_-induced toxicity.

## Discussion

Our findings in this study demonstrate that SS, the three-herb TCM formula, can ameliorate AD-related pathological symptoms. More interestingly, our results indicate that SS treatment not only ameliorated AD-related symptoms but also exhibited disease-modifying effects such as reduction of Aβ levels and Aβ amyloidosis, retardation of neuronal loss and decrease in astrocytes and microglia. Collectively, our study suggests that it is possible to alleviate symptoms and modify the disease simultaneously, and this ancient formula provides an example of such effects. Further extensive investigation is required to identify the active functional constituents as well as to elucidate the correlate molecular mechanisms, which should finally lead us to a systematic therapeutic strategy against this complicated disease.

It is characteristic of TCM that each formula comprises several herbs with different or distinct functions that work synergistically at multiple targets of a complicated disease. Our studies indicate that three herbs of SS have different therapeutic targets of AD: RT reduced Aβ generation, and AT and PRP protected neuron against Aβ-induced toxicity. RP reduced Aβ_40_ and Aβ_42_ generation in SK-N-SH-APPsw cells, showed beneficial effects in flies expressing APP/BACE but did not significantly affect locomotor function or survival in flies expressing Aβ directly. Consistently, RP treatment reduced Aβ_40_ and Aβ_42_ levels in APP/BACE transgenic fly heads, but had no significant effects in Aβ transgenic fly heads, indicating that RP reduced Aβ generation and affected the proteolysis of human APP. Furthermore, AT and PRP showed inhibitory effects on Aβ_42_ aggregation and protected primary neurons against Aβ_42_ oligomers-induced cell toxicity and apoptosis. In AD transgenic flies, AT and PRP treatment alleviated Aβ-induced premature death and motor neuron dysfunction in both Aβ transgenic flies and APP/BACE flies, suggesting that AT and PRP protect neurons through other mechanisms but may not affect APP cleavage or processing. Unfortunately, the functional active constituents of each herb are unclear in the present study. Obviously, further elucidation of the detailed molecular mechanisms should provide more efficient screening models to identify those functional constituents in each herb. Furthermore, it is of note that SS treatment improved locomotor activity and prolonged lifespan in AD *Drosophila* models, which makes it possible to quickly identify/verify functional constituents of SS in the near future. On the other hand, SS is one of many TCM formulae documented against memory loss and cognitive impairment. We must evaluate other formulae using current molecular, cellular and animal models as performed in this study, which may eventually lead to a more effective treatment against AD.

TCMs have been orally administered as decoctions, such as this formula for the so-called Smart Soup. Recently, CFDA-approved single-herb granules have been widely used due to their ease of large-scale industry production with stringent manufacturing protocols, better quality control with well-documented chemical fingerprints, and reproducible pharmacokinetic parameters. In our study, we found that the granule preparation (a mixture of three single-herb granules) was as effective as the traditional decoctions (the “soup” preparation), which simplifies future studies of individual herbs or their combination.

Although SS has been prescribed by Chinese medical physicians to patients with aging-related cognitive impairment, a multicenter, double-blind, randomized, placebo-controlled study should be carried out to evaluate the overall efficacies of SS against AD. Furthermore, whether SS should be prescribed alone or combined with other FDA-approved drugs should be assessed in future clinical trials. Moreover, it remains to be determined whether SS or other TCM formulae can be applied for MCI or for preventing neurodegenerative diseases.

## Supporting Information

Figure S1 **A schematic diagram of novel object recognition.**
(TIF)

Figure S2 **A sketch of the locomotor assay.**
(TIF)

Figure S3 **MS total ion current chromatograms of SS (SS-G).** Negative-ion (**A**) and positive-ion (**B**) modes were selected for TOF/MS analysis. Forty-five compound peaks were tentatively identified on the basis of mass measurements and retention times. Of the 45 peaks of SS, AT accounts for 5 of 45 peaks, PRP accounts for 8 of 45 peaks, and RP accounts for 32 of 45 peaks.
(TIF)

Figure S4 **HPLC fingerprints of four batches of SS (from bottom to top: 201102, 201205, 201301 and 201311).** The peaks are the characteristic and representative chemical constituents detected in SS. The similarity indices of four batches of samples were between 0.943 and 0.982.
(TIF)

Figure S5 **Representative raw data of the MWM search paths of SS- or vehicle-treated APP/PS1 mice or WT littermates.**
(TIF)

Figure S6 **RP reduces the Aβ generation in HEK293-APPsw cells.** Aβ_42_ (**A**) and Aβ_40_ (**B**) in HEK293-APPsw cell culture medium and cell viability (**C**) after treatment with SS, AT, PRP, RP for 8 hours, respectively (the triangle symbol indicates concentrations from high to low: 3000, 1000, 300 and 100 µg/ml for SS; 1000, 300, 100 and 30 µg/ml for AT, PRP and RP). *P<0.05, **P<0.01, ***P<0.001; DAPT, a γ-secretase inhibitor.
(TIF)

Figure S7 **SS and RP do not alter APP expression in APP/BACE transgenic** ***Drosophila*** **.** Western blot of human APP in APP/BACE transgenic flies cultured on SS, AT, PRP or RP (2 mg/ml). Lane 1: HEK293-APPsw cell lysates; Lane 2–6: head lysates of APP/BACE flies. Full-length APP (∼110 kD), APP-CTFs (∼10–12 kD) and Appl-CTFs (∼15 kD) were detected.
(TIF)

Figure S8 **AT and PRP improves locomotor functions and prolongs lifespan of APP/BACE transgenic** ***Drosophila*** **.** APP/BACE transgenic flies were cultured on food containing different concentrations of AT, PRP (0.2, 0.6, or 2 mg/ml) or Memantine (120 µM). (**A and C**) Survival curves for flies treated with either AT, PRP or Memantine. (**B and D**) The climbing ability of flies was assayed. The values are the mean ± S.E.M. Each value represents the mean of three experiments. Mem = Memantine. *P<0.05, **P<0.01, ***P<0.001 *vs.* the control group.
(TIF)

Figure S9 **Representative Western blots and Atomic Force Microscope (AFM) for Aβ_42_ oligomers.** (**A**) Representative western blots of Aβ_42_ monomers and oligomers separated by SDS-PAGE using 16% tricine gel and probed with the antibody 6E10 are shown. (**B**) AFM images of Aβ_42_ monomers, oligomers and fibrils are shown. The sample was scanned with AFM analysis at 10 µM. Scale bar, 0.5 µm.
(TIF)

Table S1 **Herbal Ingredients in the SS.**
(PDF)

Table S2 **Compounds identified in SS by HPLC-TOF/MS.**
(PDF)

Table S3 **The detailed information of each mouse in each group.**
(PDF)
