# Correction: Smart Soup, a Traditional Chinese Medicine Formula, Ameliorates Amyloid Pathology and Related Cognitive Deficits

**DOI:** 10.1371/journal.pone.0237035

**Published:** 2020-08-03

**Authors:** Yujun Hou, Ying Wang, Jian Zhao, Xiaohang Li, Jin Cui, Jianqing Ding, Ying Wang, Xianglu Zeng, Yun Ling, Xiaoheng Shen, Shengdi Chen, Chenggang Huang, Gang Pei

There is a reporting error in the figure legend of Fig 1. The number of animals included in each group should read n = 8–11, as opposed to the originally reported n = 9–12. Please see the complete, correct figure caption here.

**Fig 1:**
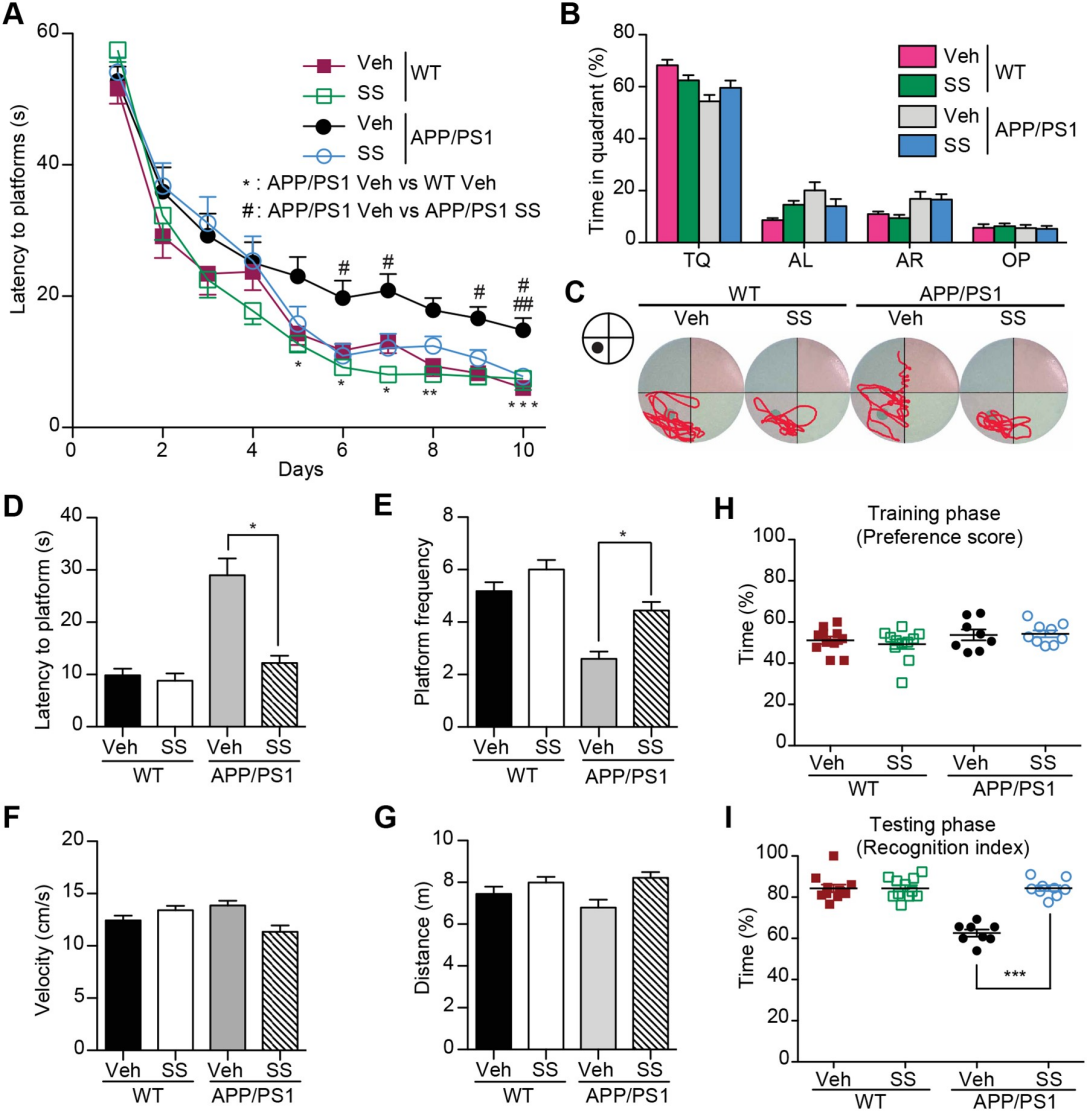
SS treatment ameliorates learning and memory impairment in Morris Water Maze and Object recognition test. **(A)** MWM test for SS and vehicle-treated APP/PS1 and WT mice. The mean escape latency was given for different test days. **(B)** The mean percent time in probe trial of MWM on day 7. TQ: Target quadrant; AL: Adjacent left; AR: Adjacent right; OP: Opposite. **(C)** Representative mice search paths from different groups. **(D and E)** The latency to target quadrant **(D)** and the frequency to pass the target position **(E)** in probe trial are shown. **(F and G)** The swimming velocity **(F)** and distance **(G)** in probe trial are shown. **(H and I)** Novel object recognition analysis. Preference scores of training phase **(H)** and Recognition Index of testing phase **(I)** during a 10-min testing phase are shown, respectively. n  =  8–11 for each group. *P<0.05, **P<0.01, ***P<0.001, #*P*<0.05, ##*P*<0.01, ###*P*<0.001.

The Cortex panels for CD11b WT Veh and APP/PS1 SS in Fig 2I appear similar. The authors have indicated that wrong cortex panel for CD11b APP/PS1 SS has been used inadvertently during the preparation of the figure. The authors have provided an updated version of Fig 2 showing the correct panel. The original images underlying the panels presented in Fig 2 have been uploaded as a supplementary file.

**Fig 2:**
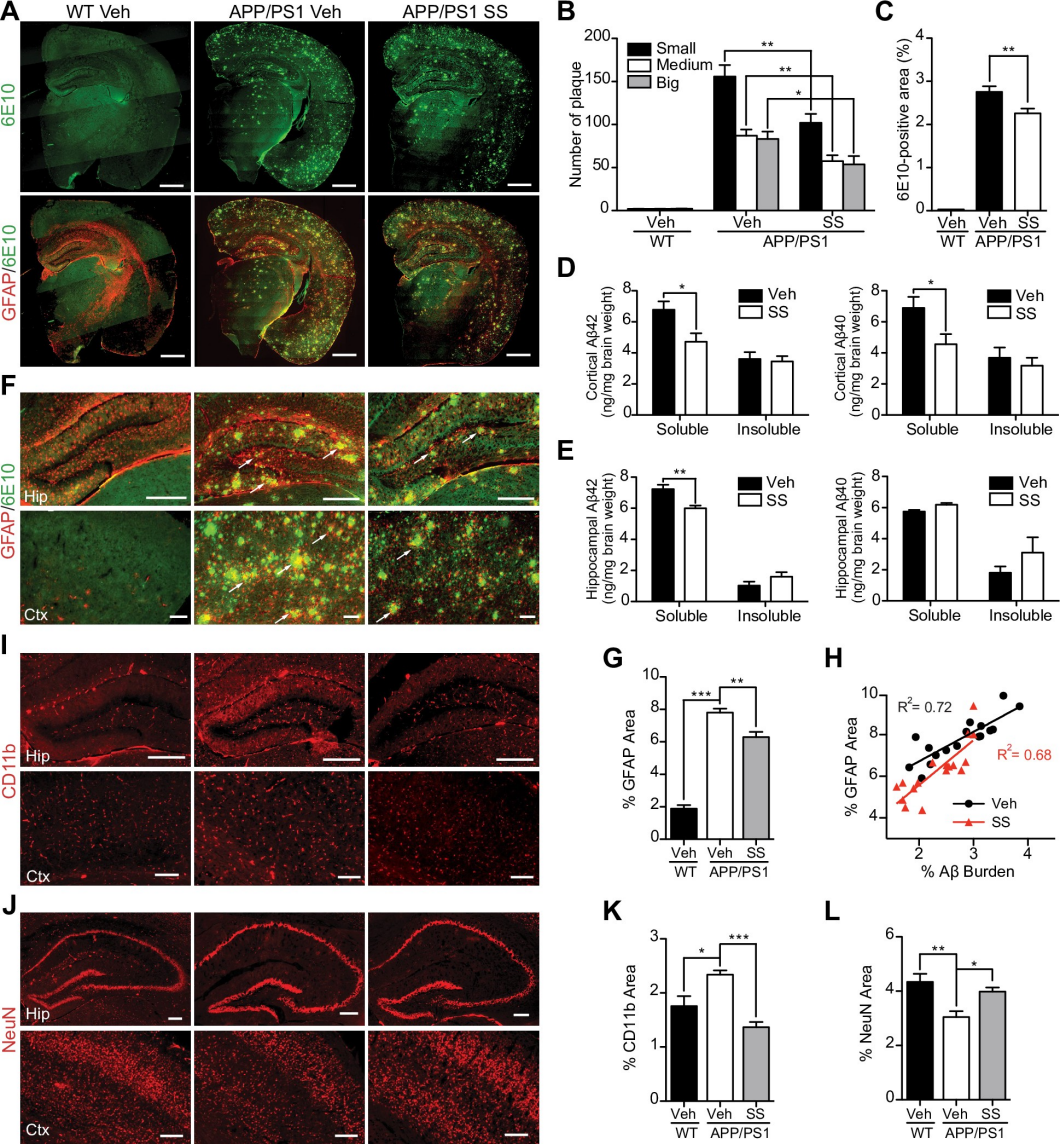
SS treatment alleviates Aβ levels and amyloid plaque burden, reduces gliosis and neuron loss in APP/PS1 mice. **(A–C)** Representative half brain sections of WT mice, vehicle or SS-treated APP/PS1 mice stained with antibody against Aβ (6E10) and double staining of GFAP and 6E10 are shown. Scale bar, 1 mm. (**B** and **C**) Quantitative analysis of the number of 6E10-positive amyloid plaques (**B**) and Aβ covered area (**C**). n  =  5 animals per group. (**D** and **E**) ELISA of soluble and insoluble Aβ_**40**_ and Aβ_**42**_ levels in cortical and hippocampal tissues of APP/PS1 mice. n  =  6 for each group. (**F**, **I** and **J**) Representative images of WT mice, vehicle- and SS- treated APP/PS1 mice hippocampus and cortex double immunostaining of GFAP and 6E10 (**F**), CD11b (**I**) and NeuN (**J**). Arrows indicate astrocytes surrounding the amyloid plaques. Scale bar, 200 µm. (**H**) Coincidence of GFAP and Aβ burden in the brains of SS-treated APP/PS1 mice (red; n  =  17) and vehicle-treated APP/PS1 mice (black; n  =  17; *P*<0.0001). (**G**, **K** and **L**) The histograms depict the mean GFAP (**G**), CD11b (**K**), and NeuN (**L**) positive area ± S.E.M. in three groups. *P<0.05, **P<0.01, ***P<0.001.

To improve the reproducibility of this study, the authors have provided additional details regarding the ingredients used to prepare the Smart Soup:

“The CFDA-approved single-herb granules of Rhizoma Acori Tatarinowii (AT), Poria cum Radix Pini (PRP) and Radix Polygalae (RP) were obtained from Tianjiang Pharmaceutical, Jiangyin, China:

AT product name: Shi Chang Pu, lot number: 1112134;

PRP product name: Fu Shen, lot number: 1103019;

RP product name: Zhi Yuan Zhi, lot number: 1102028.”

The authors have provided the underlying individual level data for their manuscript, which have been uploaded as Supporting Information Files. The original images underlying Fig 1C and Fig 7E are available from the authors upon request.

## Supporting information

S1 File Uncropped images underlying Fig 2A. (PDF)

S2 File Uncropped images underlying Fig 2F. (PDF)

S3 File Uncropped images underlying Fig 2I. (PDF)

S4 File Uncropped images underlying Fig 2J. (PDF)

S5 File Individual level data underlying Fig 1A, 1B and 1D–1I. (XLSX)

S6 File Individual level data underlying Fig 2B–2E, 2G, 2H, 2K and 2L. (XLSX)

S7 File Individual level data underlying Fig 3A–3F. (XLSX)

S8 File Individual level data underlying Fig 4A–4C. (XLSX)

S9 File Individual level data underlying Fig 5A–5D. (XLSX)

S10 File Individual level data underlying Fig 6A–6D. (XLSX)

S11 File Individual level data underlying Fig 7A–7D and 7F. (XLSX)
