# Supplementary figures and images for: Smart Soup, a Traditional Chinese Medicine Formula, Ameliorates Amyloid Pathology and Related Cognitive Deficits
Source: PLoS One. 2014 Nov 11;9(11):e111215. doi: 10.1371/journal.pone.0111215 (PMC4227681; doi:10.1371/journal.pone.0111215)

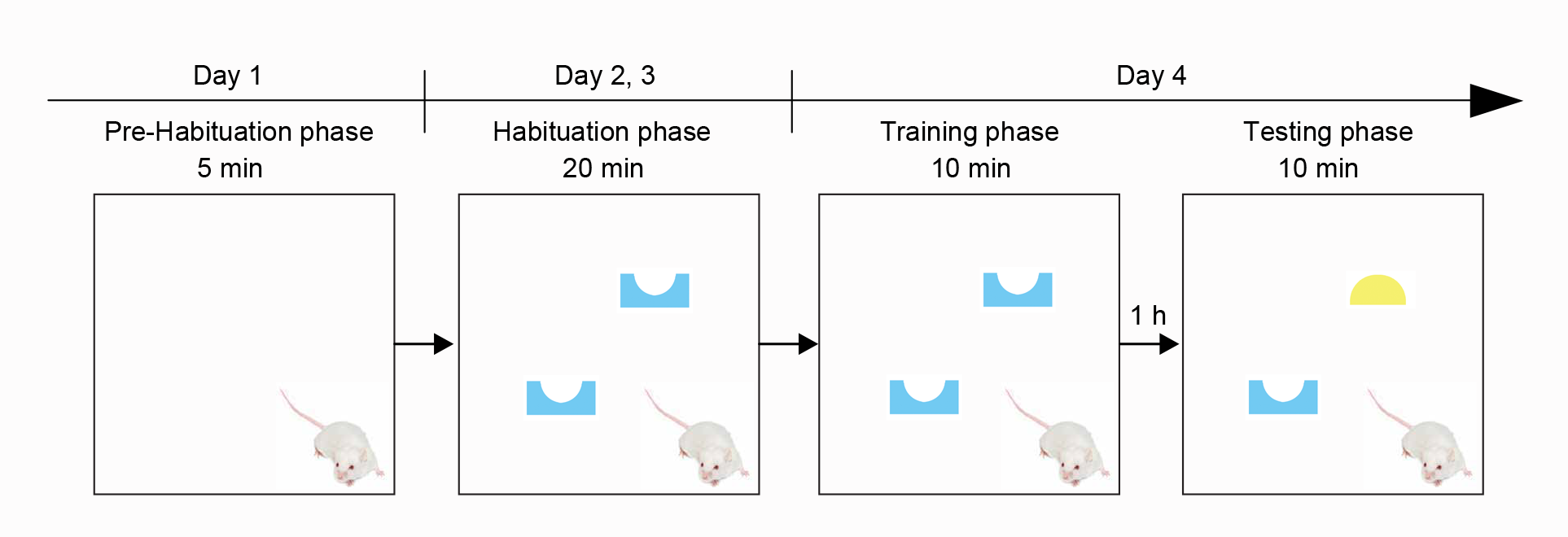

Supplement: Figure S1 — A schematic diagram of novel object recognition. (TIF) [file pone.0111215.s001.tif]

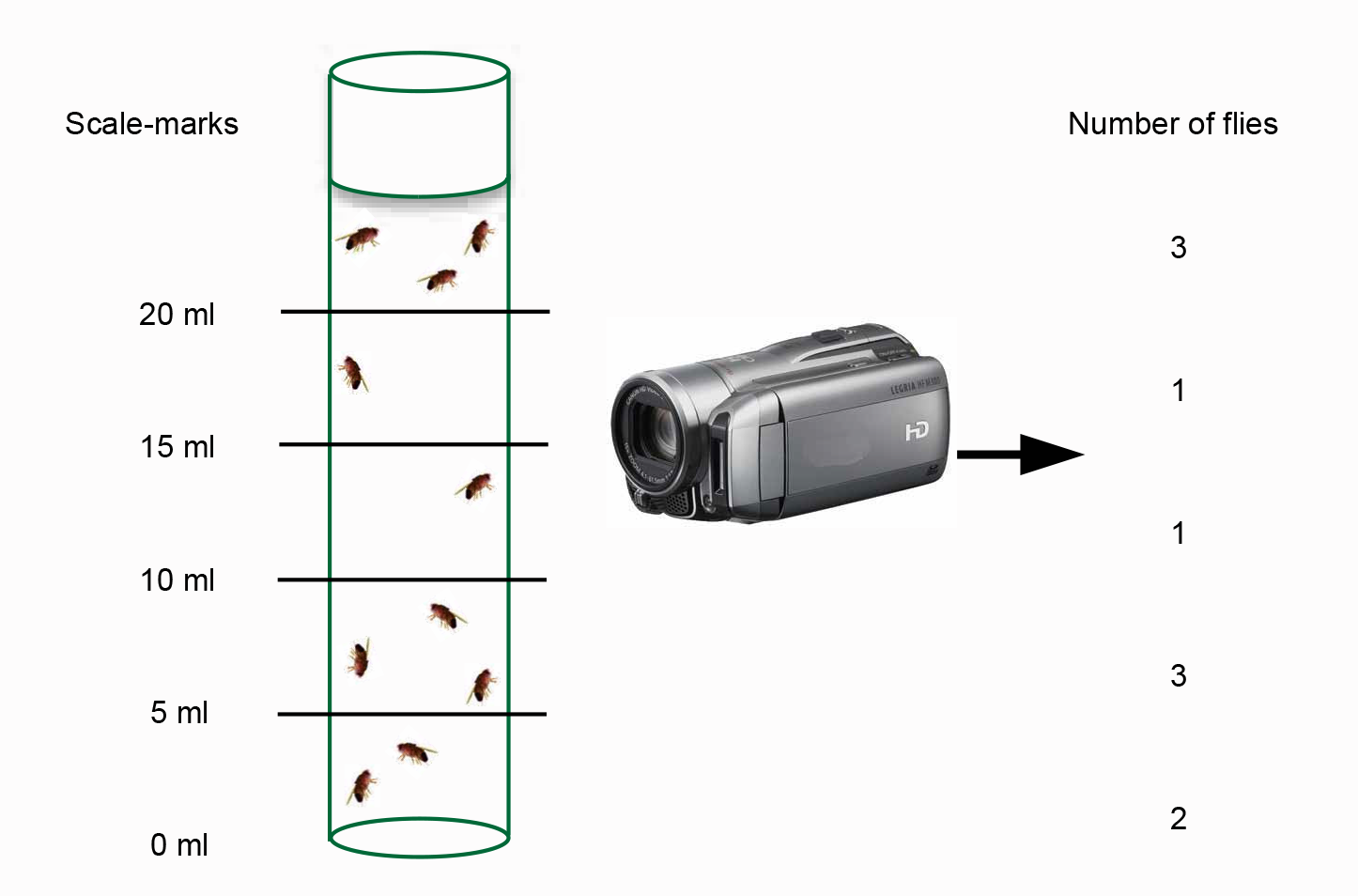

Supplement: Figure S2 — A sketch of the locomotor assay. (TIF) [file pone.0111215.s002.tif]

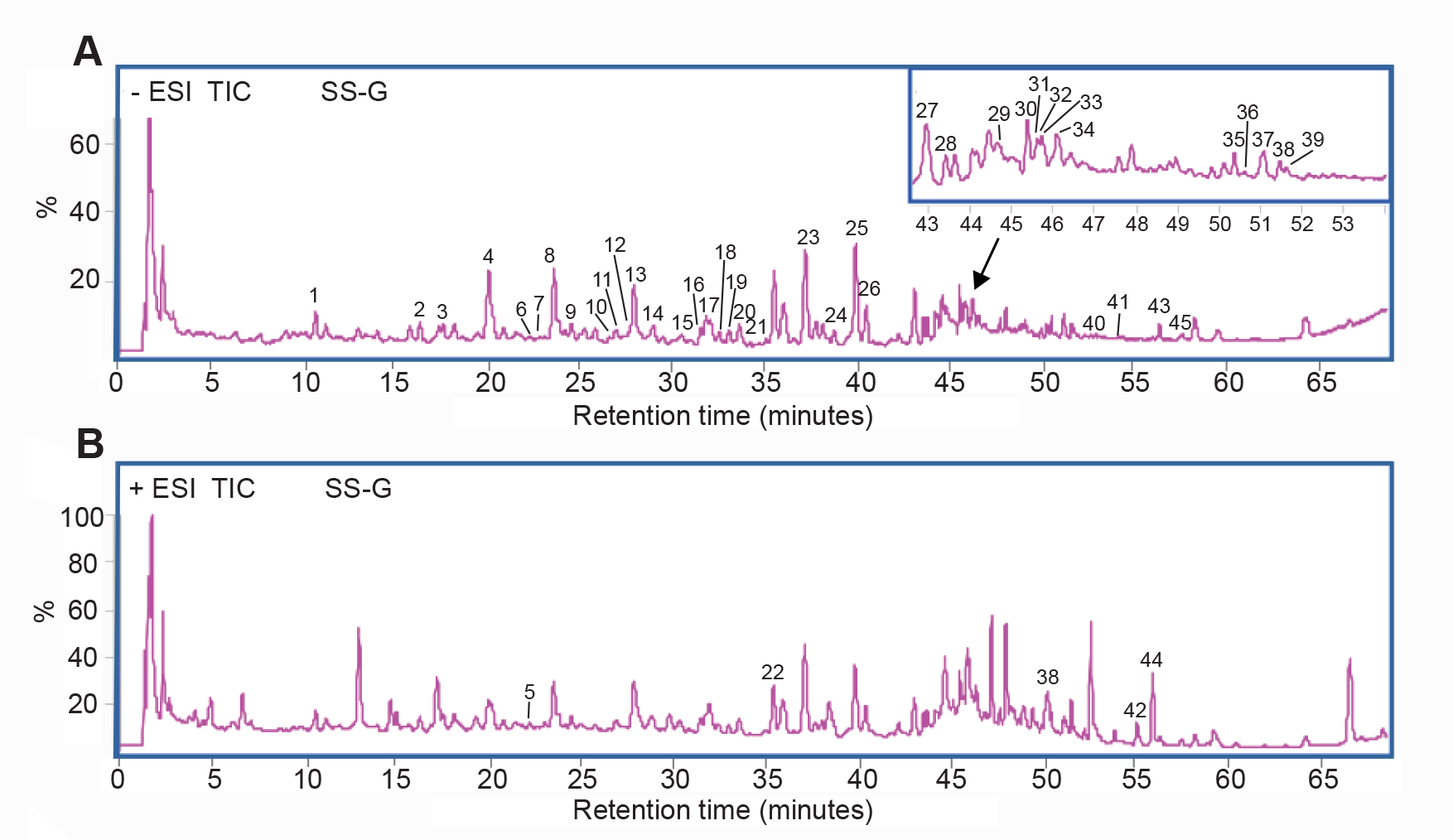

Supplement: Figure S3 — MS total ion current chromatograms of SS (SS-G). Negative-ion (A) and positive-ion (B) modes were selected for TOF/MS analysis. Forty-five compound peaks were tentatively identified on the basis of mass measurements and retention times. Of the 45 peaks of SS, AT accounts for 5 of 45 peaks, PRP accounts for 8 of 45 peaks, and RP accounts for 32 of 45 peaks. (TIF) [file pone.0111215.s003.tif]

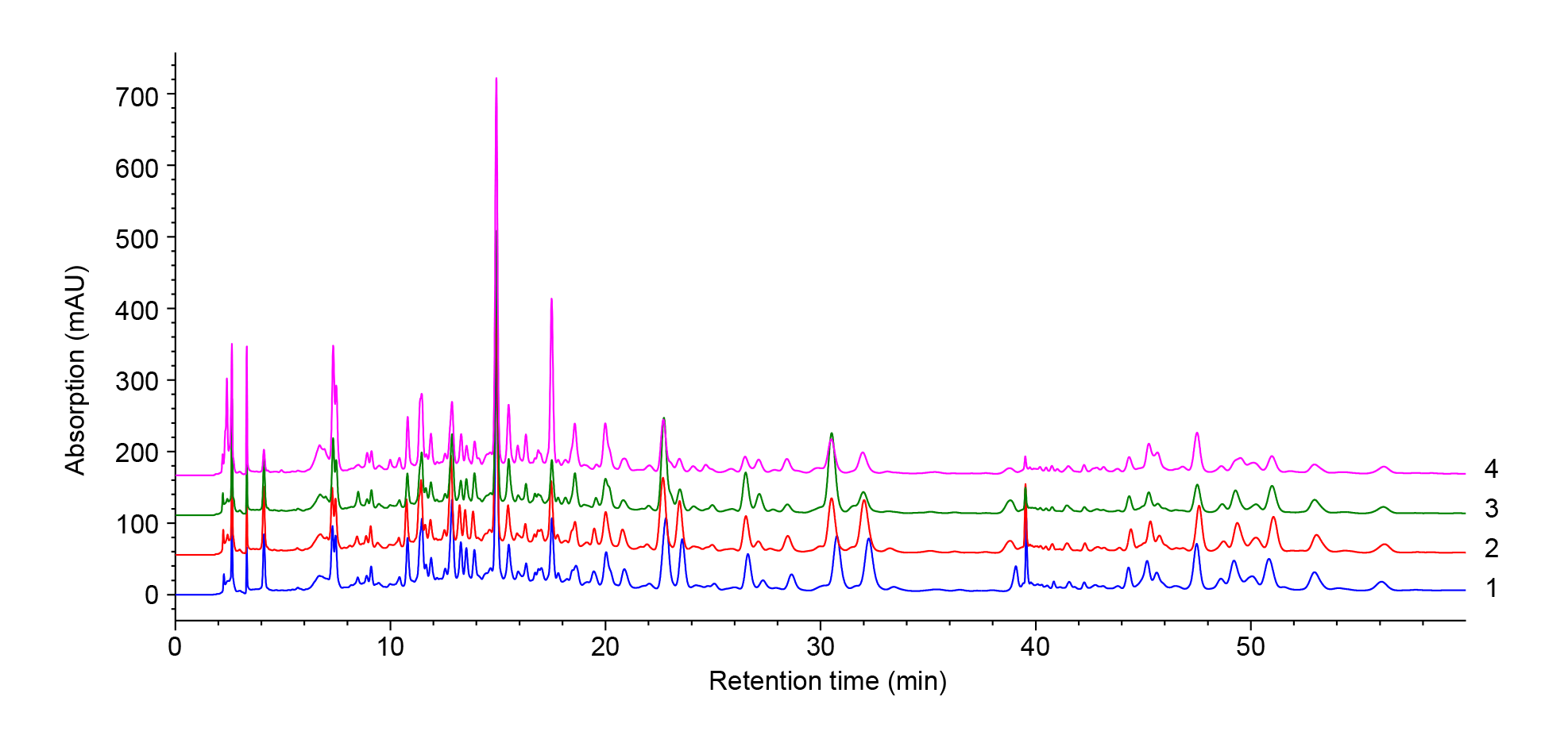

Supplement: Figure S4 — HPLC fingerprints of four batches of SS (from bottom to top: 201102, 201205, 201301 and 201311). The peaks are the characteristic and representative chemical constituents detected in SS. The similarity indices of four batches of samples were between 0.943 and 0.982. (TIF) [file pone.0111215.s004.tif]

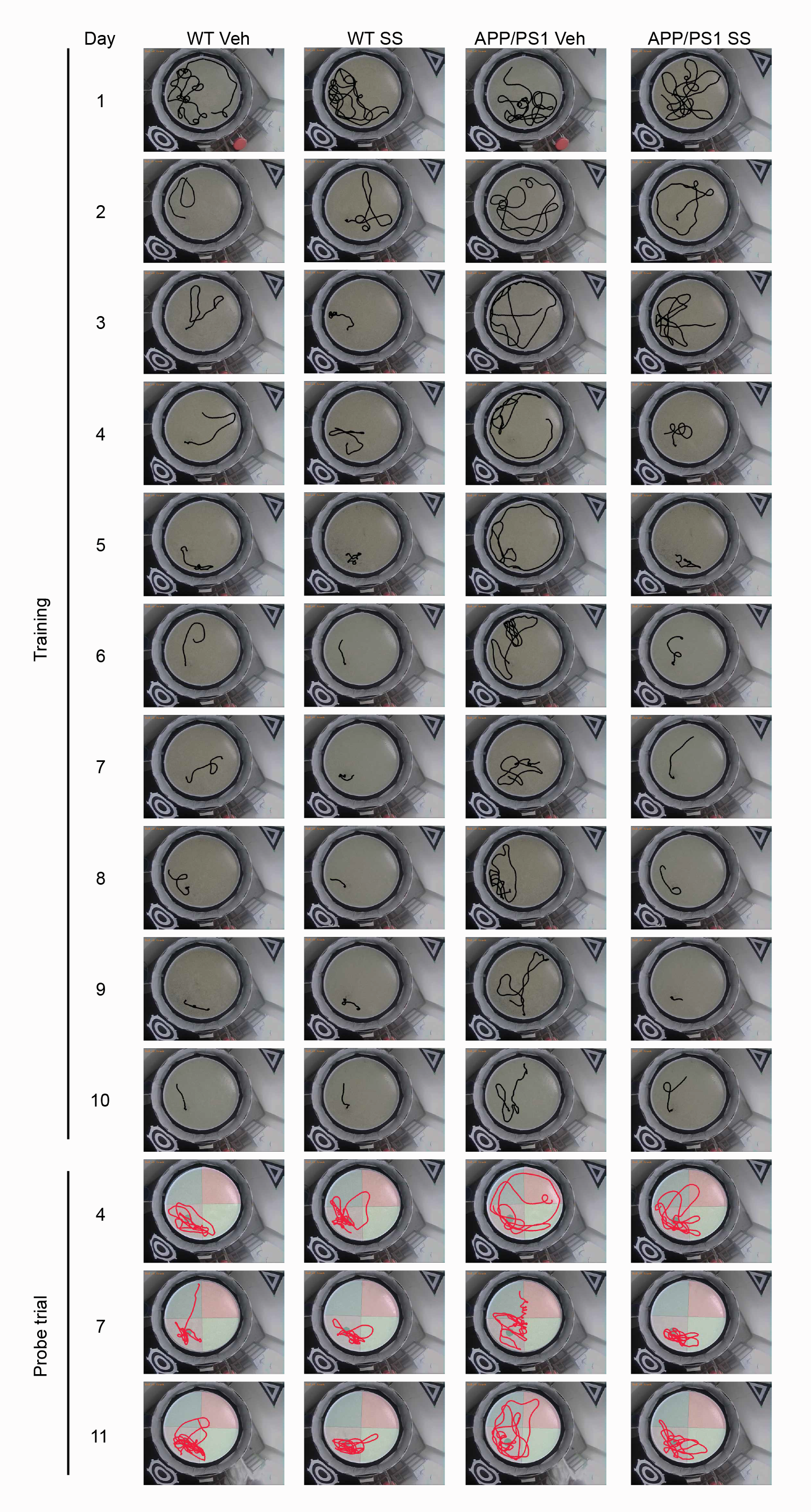

Supplement: Figure S5 — Representative raw data of the MWM search paths of SS- or vehicle-treated APP/PS1 mice or WT littermates. (TIF) [file pone.0111215.s005.tif]

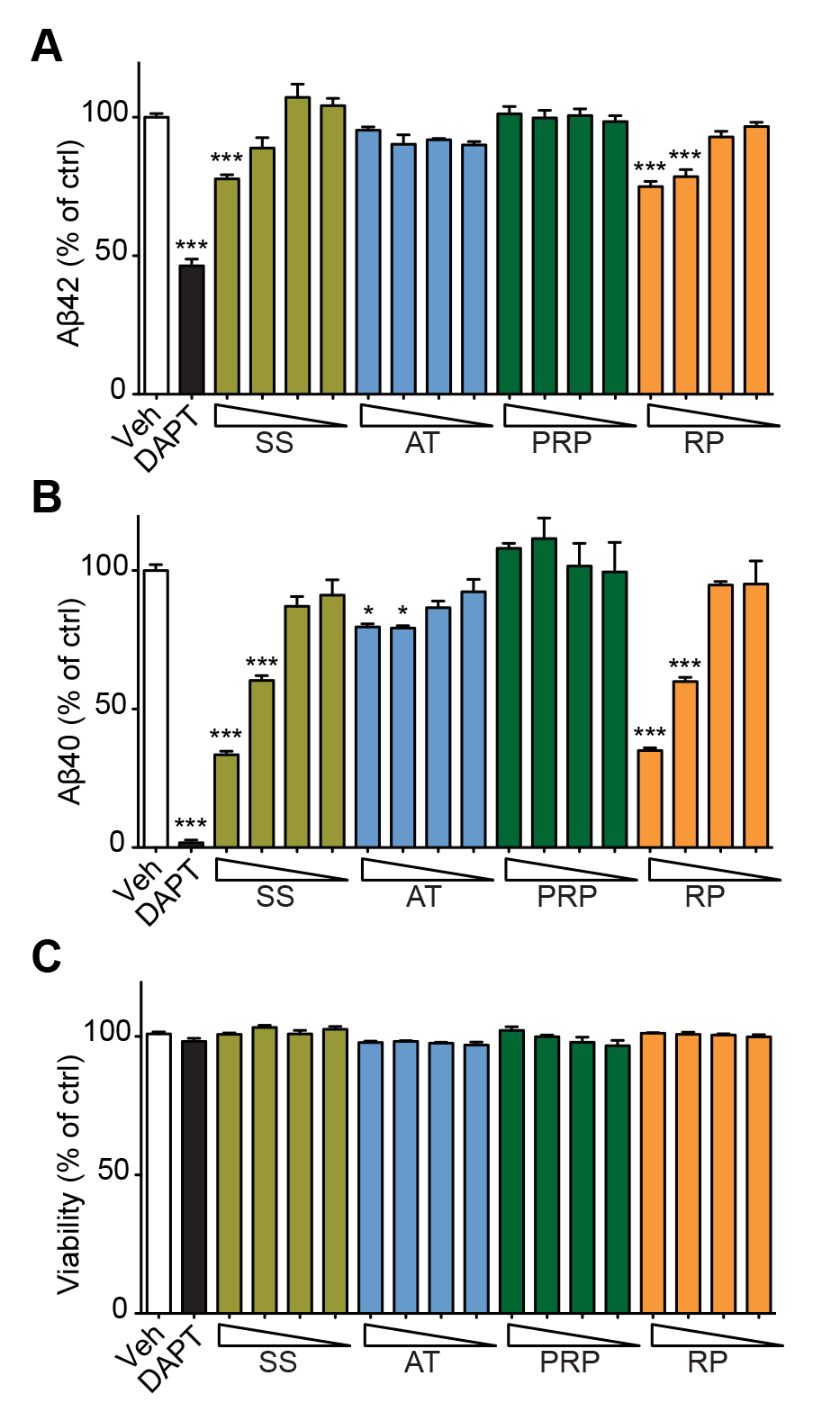

Supplement: Figure S6 — RP reduces the Aβ generation in HEK293-APPsw cells. Aβ42 (A) and Aβ40 (B) in HEK293-APPsw cell culture medium and cell viability (C) after treatment with SS, AT, PRP, RP for 8 hours, respectively (the triangle symbol indicates concentrations from high to low: 3000, 1000, 300 and 100 µg/ml for SS; 1000, 300, 100 and 30 µg/ml for AT, PRP and RP). *P<0.05, **P<0.01, ***P<0.001; DAPT, a γ-secretase inhibitor. (TIF) [file pone.0111215.s006.tif]

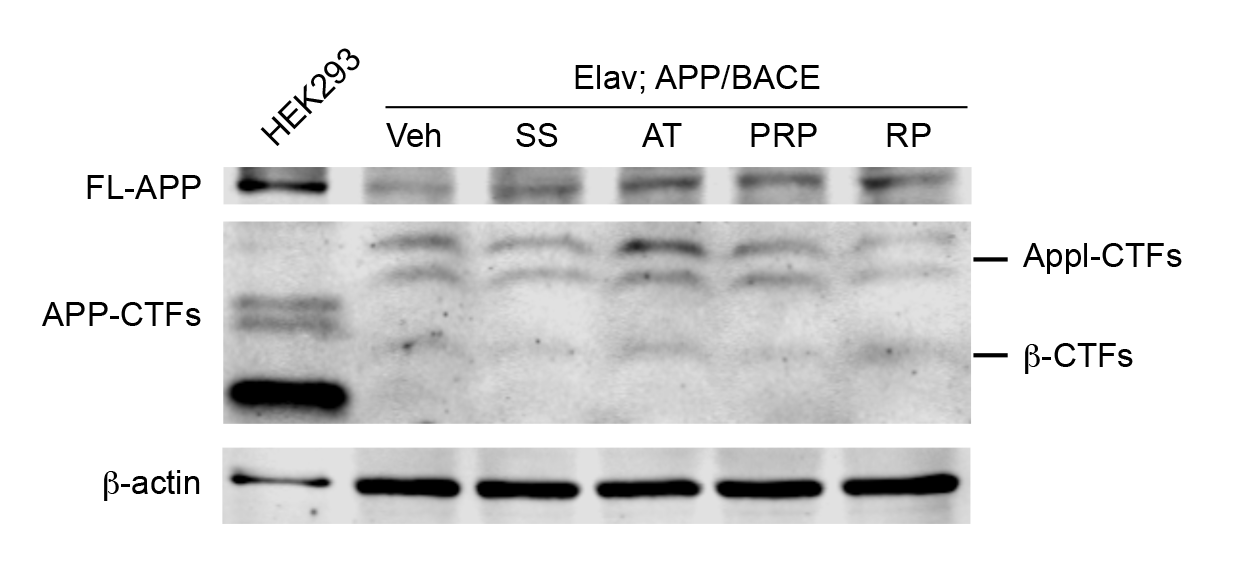

Supplement: Figure S7 — SS and RP do not alter APP expression in APP/BACE transgenic Drosophila . Western blot of human APP in APP/BACE transgenic flies cultured on SS, AT, PRP or RP (2 mg/ml). Lane 1: HEK293-APPsw cell lysates; Lane 2–6: head lysates of APP/BACE flies. Full-length APP (∼110 kD), APP-CTFs (∼10–12 kD) and Appl-CTFs (∼15 kD) were detected. (TIF) [file pone.0111215.s007.tif]

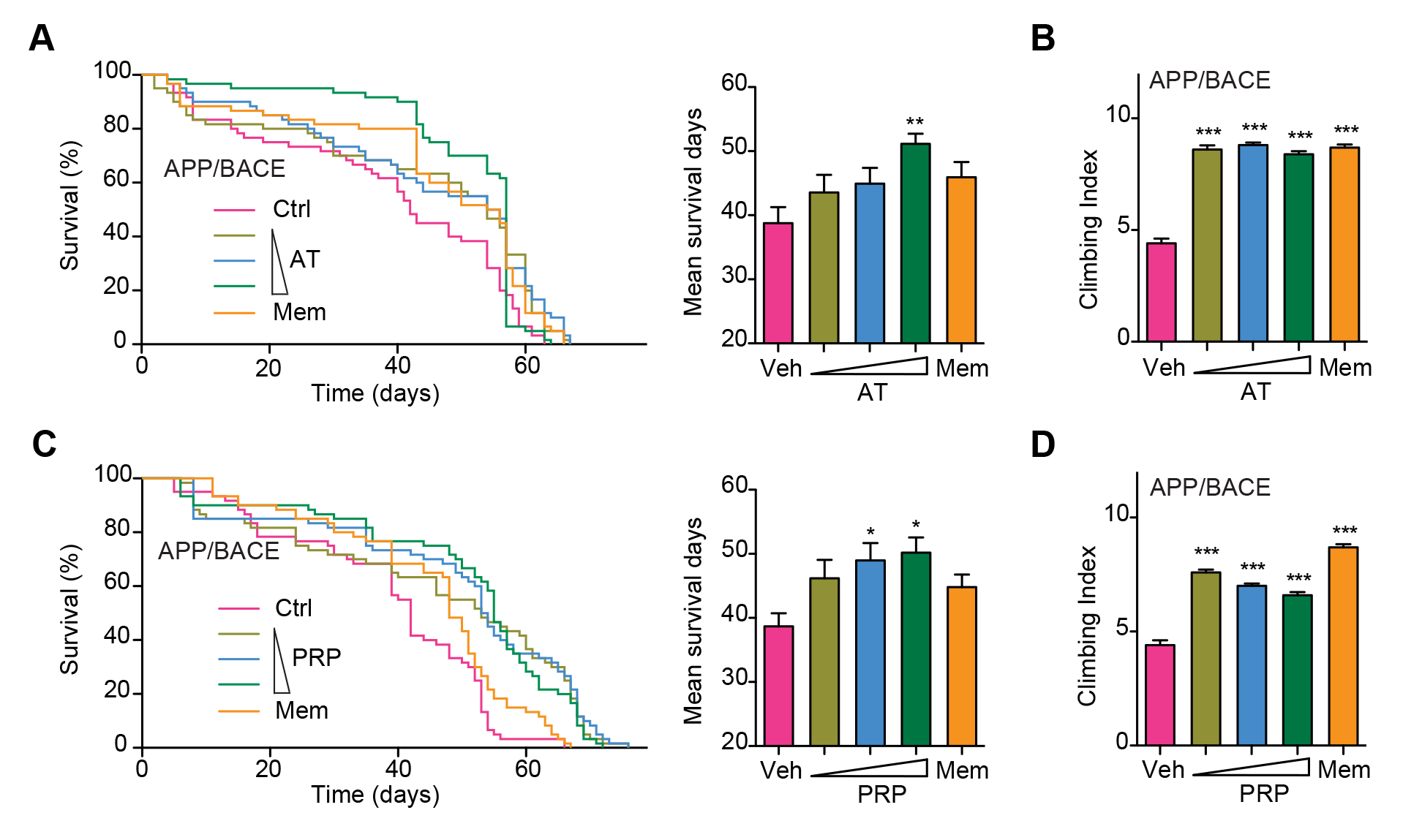

Supplement: Figure S8 — AT and PRP improves locomotor functions and prolongs lifespan of APP/BACE transgenic Drosophila . APP/BACE transgenic flies were cultured on food containing different concentrations of AT, PRP (0.2, 0.6, or 2 mg/ml) or Memantine (120 µM). (A and C) Survival curves for flies treated with either AT, PRP or Memantine. (B and D) The climbing ability of flies was assayed. The values are the mean ± S.E.M. Each value represents the mean of three experiments. Mem = Memantine. *P<0.05, **P<0.01, ***P<0.001 vs. the control group. (TIF) [file pone.0111215.s008.tif]

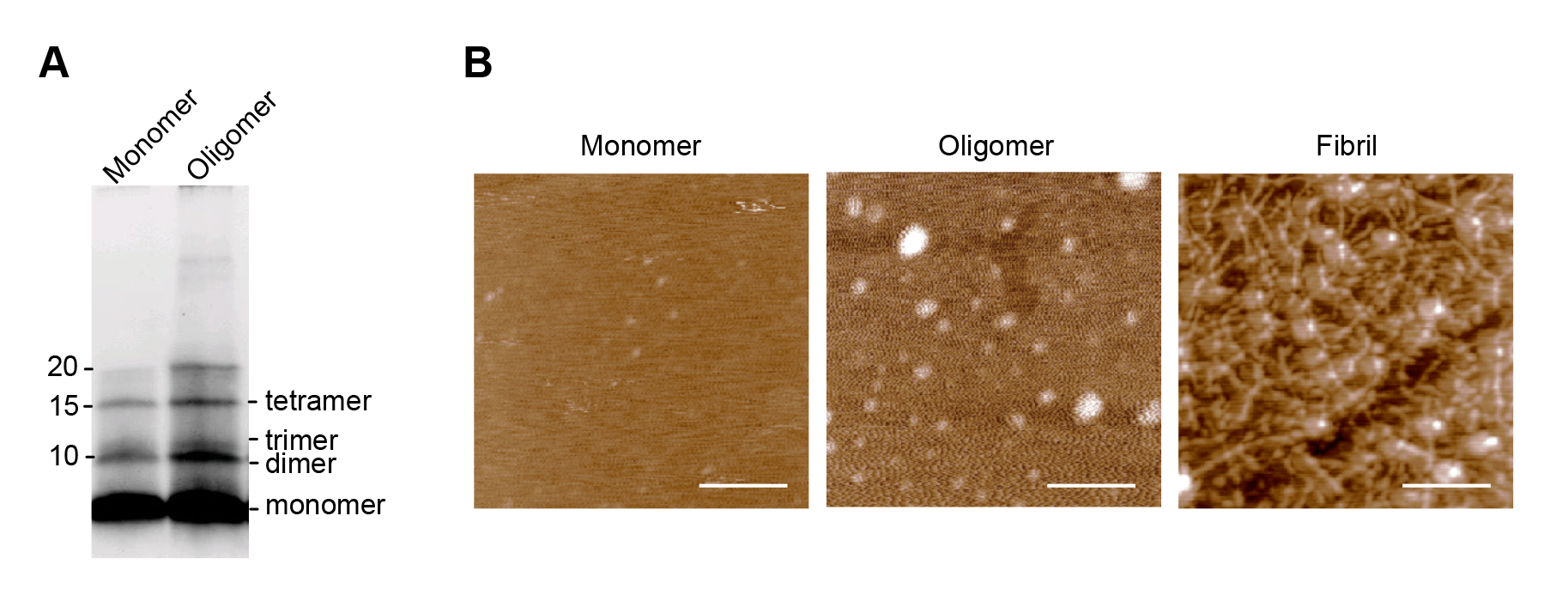

Supplement: Figure S9 — Representative Western blots and Atomic Force Microscope (AFM) for Aβ42 oligomers. (A) Representative western blots of Aβ42 monomers and oligomers separated by SDS-PAGE using 16% tricine gel and probed with the antibody 6E10 are shown. (B) AFM images of Aβ42 monomers, oligomers and fibrils are shown. The sample was scanned with AFM analysis at 10 µM. Scale bar, 0.5 µm. (TIF) [file pone.0111215.s009.tif]
