# Supplementary material for: Smart Soup, a Traditional Chinese Medicine Formula, Ameliorates Amyloid Pathology and Related Cognitive Deficits
Source: PLoS One. 2014 Nov 11;9(11):e111215. doi: 10.1371/journal.pone.0111215 (PMC4227681; doi:10.1371/journal.pone.0111215)
Supplement: Table S1 — Herbal Ingredients in the SS. (PDF) [file pone.0111215.s010.pdf]

Table S1. Herbal Ingredients in the SS.

Pharmaceutical Name	Latin Name	English Name	Traditional Usage (for)	Recent Studies
Rhizoma Acori Tatarinowii	*Acorus tatarinowii* Schott.	Grassleaf Sweetflag Rhizome	• Chronic gastritis, feeling of oppression<br>• Delirium, deafness, tinnitus<br>• Rheumatic arthritis, lumbago	• Show neuroprotective effects against excitotoxic neural death<br>• Attenuate learning and memory deficits [7]
Poria cum Radix Pini	*Poria cocos* (Schw.) Wolf	Tuckhoe with pine	• Edema, dysuria<br>• Palpitation, insomnia	• Possess sedative activity [8]
Radix Polygalae	*Polygala tenuifolia* Willd.	Thinleaf Milkwort Root	• Irritability, apprehension, palpitation, insomnia, excessive dreams<br>• Convulsion, insanity<br>• Productive cough with thick sputum<br>• Skin infection, acute mastitis and breast nodules	• Show neuroprotective effects [10,11]<br>• Enhance cognitive functions in elderly individuals and show memory enhancement in healthy adults [12,13]
