# Supplementary material for: Smart Soup, a Traditional Chinese Medicine Formula, Ameliorates Amyloid Pathology and Related Cognitive Deficits
Source: PLoS One. 2014 Nov 11;9(11):e111215. doi: 10.1371/journal.pone.0111215 (PMC4227681; doi:10.1371/journal.pone.0111215)
Supplement: Table S2 — Compounds identified in SS by HPLC-TOF/MS. (PDF) [file pone.0111215.s011.pdf]

Table S2. Compounds identified in SS by HPLC-TOF/MS.

No	t_R (min)	Molecular formula	Ion mode	Mass (m/z) Measured (m/z)	Calculated (m/z)	Error (ppm)	Identification	Source[a]
1	10.985	$C_{23}H_{32}O_{15}$	$[M-H]^-$	547.1687	547.1668	3.47	Sibiricose A1	RP
2	16.246	$C_{24}H_{26}O_{14}$	$[M-H]^-$	537.1223	537.125	-5.03	Sibiricaxanthone A	RP
3	17.399	$C_{25}H_{28}O_{15}$	$[M-H]^-$	567.138	567.1355	4.41	Polygalaxanthone III	RP
4	20.037	$C_{30}H_{36}O_{17}$	$[M-H]^-$	667.1686	667.188	0.89	Tenuifoliside B	RP
5	21.911	$C_{12}H_{16}O_4$	$[M+H]^+$	225.1126	225.1121	2.22	Acoramone	AT
6	22.476	$C_{26}H_{30}O_{15}$	$[M-H]^-$	581.1558	581.1512	7.92	Polygalaxanthone V	RP
7	22.893	$C_{27}H_{32}O_{16}$	$[M-H]^-$	611.1588	611.1618	-4.91	Polygalaxanthone VII	RP
8	23.519	$C_{34}H_{42}O_{19}$	$[M-H]^-$	753.225	753.2248	0.26	3, 6'-disinapoyl sucrose (DSS)	RP
9	24.363	$C_{33}H_{40}O_{18}$	$[M-H]^-$	723.2136	723.2142	-0.83	Sucrose 10	RP
10	26.668	$C_{30}H_{36}O_{16}$	$[M-H]^-$	651.1942	651.1931	1.69	Sucrose 8	RP
11	26.752	$C_{30}H_{36}O_{16}$	$[M-H]^-$	651.1951	651.1931	3.07	3'-sinapoyl-6-benzoyl sucrose	RP
12	27.536	$C_{66}H_{84}O_{38}$	$[M-H]^-$	1483.459	1483.457	1.34	Tenuifoliose G	RP
13	27.804	$C_{31}H_{38}O_{17}$	$[M-H]^-$	681.2054	681.2036	2.64	Tenuifoliside A	RP
14	29.324	$C_{34}H_{42}O_{18}$	$[M-H]^-$	737.232	737.2298	2.98	Sucrose 18	RP
15	30.476	$C_{35}H_{44}O_{19}$	$[M-H]^-$	767.2406	767.2404	0.26	Tenuifoliside C	RP
16	31.545	$C_{68}H_{86}O_{39}$	$[M-H]^-$	1525.473	1525.467	3.67	Tenuifoliose F	RP
17	31.645	$C_{67}H_{84}O_{38}$	$[M-H]^-$	1495.456	1495.457	-0.47	Tenuifoliose L	RP
18	32.747	$C_{57}H_{70}O_{32}$	$[M-H]^-$	1265.382	1265.378	3	Tenuifoliose K	RP
19	33.559	$C_{58}H_{72}O_{33}$	$[M-H]^-$	1295.394	1295.388	4.71	Tenuifoliose C	RP
20	33.683	$C_{35}H_{42}O_{19}$	$[M-H]^-$	765.223	765.2248	-2.35	Sucrose 12	RP
21	34.117	$C_{59}H_{74}O_{34}$	$[M-H]^-$	1325.405	1325.399	4.83	Tenuifoliose P	RP
22	34.561	$C_{10}H_{12}O_4$	$[M+H]^+$	197.0817	197.0808	4.57	Asaronaldehyde	AT
23	37.758	$C_{53}H_{84}O_{24}$	$[M-H]^-$	1103.525	1103.528	-2.81	Polygalasaponin XXVIII	RP
24	39.244	$C_{61}H_{76}O_{35}$	$[M-H]^-$	1367.414	1367.409	3.07	Tenuifoliose O	RP
25	39.651	$C_{61}H_{74}O_{34}$	$[M-H]^-$	1349.395	1349.399	-2.59	Tenuifoliose H	RP
26	40.697	$C_{69}H_{102}O_{30}$	$[M-H]^-$	1409.414	1409.411	2.34	Onjisaponin Y	RP
27	43.055	$C_{59}H_{72}O_{33}$	$[M-H]^-$	1307.382	1307.388	-4.82	Tenuifoliose I	RP
28	43.456	$C_{60}H_{74}O_{34}$	$[M-H]^-$	1337.394	1337.399	-3.81	Tenuifoliose B	RP
29	44.873	$C_{76}H_{114}O_{37}$	$[M-H]^-$	1617.693	1617.697	-2.04	Onjisaponin R	RP
30	45.508	$C_{77}H_{116}O_{37}$	$[M-H]^-$	1631.715	1631.712	1.72	Onjisaponin O	RP
31	45.608	$C_{75}H_{112}O_{35}$	$[M-H]^-$	1571.687	1571.691	-2.8	Onjisaponin B	RP
32	45.741	$C_{79}H_{118}O_{38}$	$[M-H]^-$	1673.733	1673.723	6.27	Tenuifoside A	RP
33	45.908	$C_{71}H_{106}O_{33}$	$[M-H]^-$	1485.659	1485.654	2.83	Onjisaponin E	RP
34	46.142	$C_{75}H_{112}O_{36}$	$[M-H]^-$	1587.695	1587.695	-0.25	Onjisaponin F	RP
35	50.368	$C_{30}H_{44}O_5$	$[M-H]^-$	483.3106	483.3116	-2.07	Poricoic acid B	PRP
36	50.936	$C_{31}H_{46}O_5$	$[M-H]^-$	497.3296	497.3272	4.83	Poricoic acid A	PRP
37	51.065	$C_{30}H_{46}O_5$	$[M-H]^-$	485.3248	485.3272	-4.94	Poricoic acid G	PRP
38	51.394	$C_{12}H_{16}O_3$	$[M+H]^+$	209.1177	209.1172	2.39	α-Asarone	AT
39	51.482	$C_{30}H_{46}O_4$	$[M-H]^-$	469.3284	469.3284	0	3β, 16α-Dihydroxy-lanosta-7, 9 (11), 24-trien-21-oic acid	PRP
40	53.217	$C_{32}H_{50}O_5$	$[M-H]^-$	513.3574	513.3585	-2.14	3-O-acetyl-16α-hydroxytrametenolic acid	PRP
41	53.975	$C_{33}H_{52}O_5$	$[M-H]^-$	527.3712	527.3742	-5.69	Pachymic acid	PRP
42	54.938	$C_{24}H_{32}O_6$	$[M+Na]^+$	439.2114	439.2041	-6.14	Diasarone I	AT
43	56.314	$C_{30}H_{46}O_3$	$[M-H]^-$	453.3375	453.3374	0.22	Dehydrotrametenolic acid	PRP
44	56.409	$C_{36}H_{48}O_9$	$[M+Na]^+$	647.3214	647.3191	3.55	Tatarinan A	AT
45	57.115	$C_{30}H_{48}O_3$	$[M-H]^-$	455.3555	455.3531	5.27	Trametenolic acid	PRP

[a]RP = Radix Polygalae; AT = Rhizoma Acori Tatarinowii; PRP = Poria cum Radix Pini.
