# Supplementary material for: Smart Soup, a Traditional Chinese Medicine Formula, Ameliorates Amyloid Pathology and Related Cognitive Deficits
Source: PLoS One. 2014 Nov 11;9(11):e111215. doi: 10.1371/journal.pone.0111215 (PMC4227681; doi:10.1371/journal.pone.0111215)
Supplement: Table S3 — The detailed information of each mouse in each group. (PDF) [file pone.0111215.s012.pdf]

Table S3. The detailed information of each mouse in each group.

Group	Birthday	Before drug	Age when MWM			After MWM	Age when Perfusion			Perfusion
		Weight (g)	Days	Weeks	Months	Weight (g)	Days	Weeks	Months	Weight (g)
WT Veh	2010-9-15	30.4	288	41.1	9.3	30.2	447	63.9	14.4	30.8
	2010-10-1	27.9	272	38.9	8.8	28.1	431	61.6	13.9	27.9
	2010-9-15	26.2	288	41.1	9.3	26.2	447	63.9	14.4	26.3
	2010-9-15	27.6	288	41.1	9.3	27.8	447	63.9	14.4	27.8
	2010-9-15	22.4	288	41.1	9.3	22.2	447	63.9	14.4	20.9
	2010-9-26	26.2	277	39.6	8.9	26.8	436	62.3	14.1	27.1
	2010-9-13	24.6	290	41.4	9.4	24.1	449	64.1	14.5	24.4
	2010-9-13	24.0	290	41.4	9.4	24.0	449	64.1	14.5	24.3
	2010-9-26	27.3	277	39.6	8.9	27.5	436	62.3	14.1	27.1
	2010-9-26	26.1	277	39.6	8.9	26.3	436	62.3	14.1	28.0
	2010-9-15	28.0	288	41.1	9.3	27.6	447	63.9	14.4	28.6
Average of WT Veh		26.4	284	40.6	9.2	26.4	443	63.3	14.3	26.7
WT SS	2010-10-1	27.3	272	38.9	8.8	27.2	431	61.6	13.9	27.5
	2010-9-15	28.1	288	41.1	9.3	27.6	447	63.9	14.4	28.3
	2010-9-13	25.9	290	41.4	9.4	26.1	449	64.1	14.5	26.0
	2010-9-15	30.3	288	41.1	9.3	29.7	447	63.9	14.4	31.9
	2010-9-15	26.1	288	41.1	9.3	26.3	447	63.9	14.4	26.9
	2010-9-15	23.9	288	41.1	9.3	24.2	447	63.9	14.4	25.5
	2010-9-26	22.2	277	39.6	8.9	21.9	436	62.3	14.1	21.7
	2010-9-26	24.9	277	39.6	8.9	24.8	436	62.3	14.1	25.0
	2010-9-15	29.0	288	41.1	9.3	28.5	447	63.9	14.4	30.3
	2010-9-15	30.6	288	41.1	9.3	31.8	447	63.9	14.4	32.7
Average of WT SS		26.8	284	40.6	9.2	26.8	443	63.3	14.3	27.6
APP/PS1 Veh	2010-9-15	28.1	288	41.1	9.3	27.4	447	63.9	14.4	29.8
	2010-10-1	24.3	272	38.9	8.8	24.5	431	61.6	13.9	24.2
	2010-10-1	26.6	272	38.9	8.8	26.8	431	61.6	13.9	27.2
	2010-9-15	26.2	288	41.1	9.3	26.4	447	63.9	14.4	26.4
	2010-9-15	31.2	288	41.1	9.3	30.8	447	63.9	14.4	33.0
	2010-9-13	27.5	290	41.4	9.4	27.9	449	64.1	14.5	28.3
	2010-9-15	27.5	288	41.1	9.3	27.9	447	63.9	14.4	26.4
	2010-9-15	29.6	288	41.1	9.3	30.1	447	63.9	14.4	29.8
	2010-9-15	26.8	288	41.1	9.3	27.3	447	63.9	14.4	26.9
	2010-9-26	26.1	277	39.6	8.9	26.0	436	62.3	14.1	26.2
Average of APP/PS1 Veh		27.4	284	40.6	9.2	27.5	443	63.3	14.3	27.8
APP/PS1 SS	2010-9-15	30.3	288	41.1	9.3	30.4	447	63.9	14.4	30.6
	2010-10-1	25.9	272	38.9	8.8	26.1	431	61.6	13.9	25.8
	2010-10-1	29.1	272	38.9	8.8	28.9	431	61.6	13.9	29.1
	2010-10-1	22.5	272	38.9	8.8	22.8	431	61.6	13.9	22.9
	2010-9-13	30.2	290	41.4	9.4	30.8	449	64.1	14.5	30.5
	2010-9-13	25.9	290	41.4	9.4	26.7	449	64.1	14.5	26.2
	2010-9-15	25.2	288	41.1	9.3	25.4	447	63.9	14.4	25.9
	2010-9-15	28.0	288	41.1	9.3	28.1	447	63.9	14.4	28.2
	2010-9-29	27.4	274	39.1	8.8	27.7	433	61.9	14.0	27.5
Average of APP/PS1 SS		27.2	282	40.2	9.1	27.4	441	62.9	14.2	27.4
