# Supplementary figures and images for: Correction: Smart Soup, a Traditional Chinese Medicine Formula, Ameliorates Amyloid Pathology and Related Cognitive Deficits
Source: PLoS One. 2020 Aug 3;15(8):e0237035. doi: 10.1371/journal.pone.0237035 (PMC7398536; doi:10.1371/journal.pone.0237035)

Figure 2A original images

WT Veh 6E10

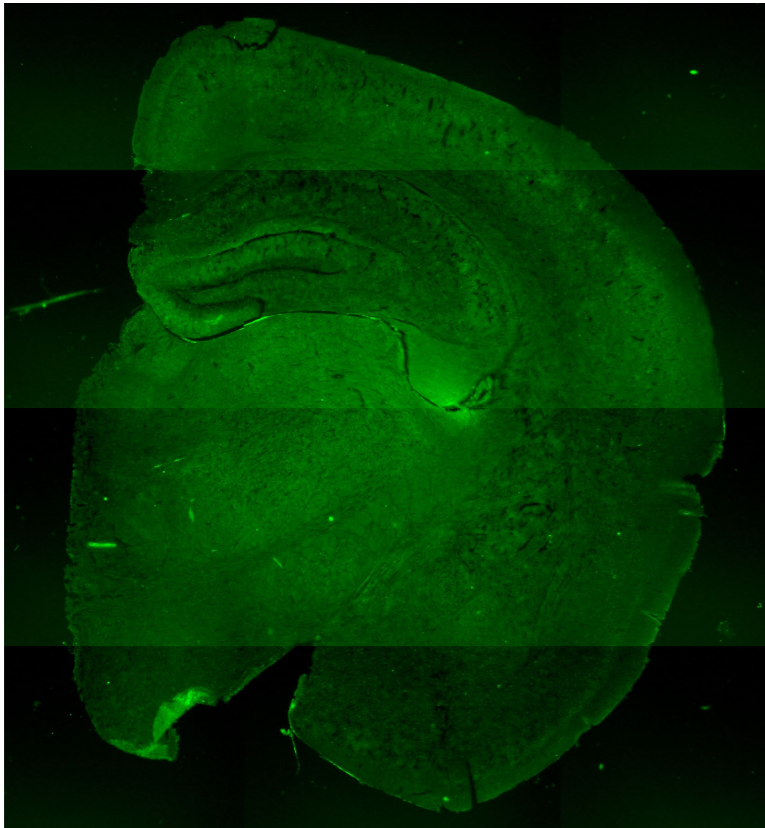

APP/PS1 Veh 6E10

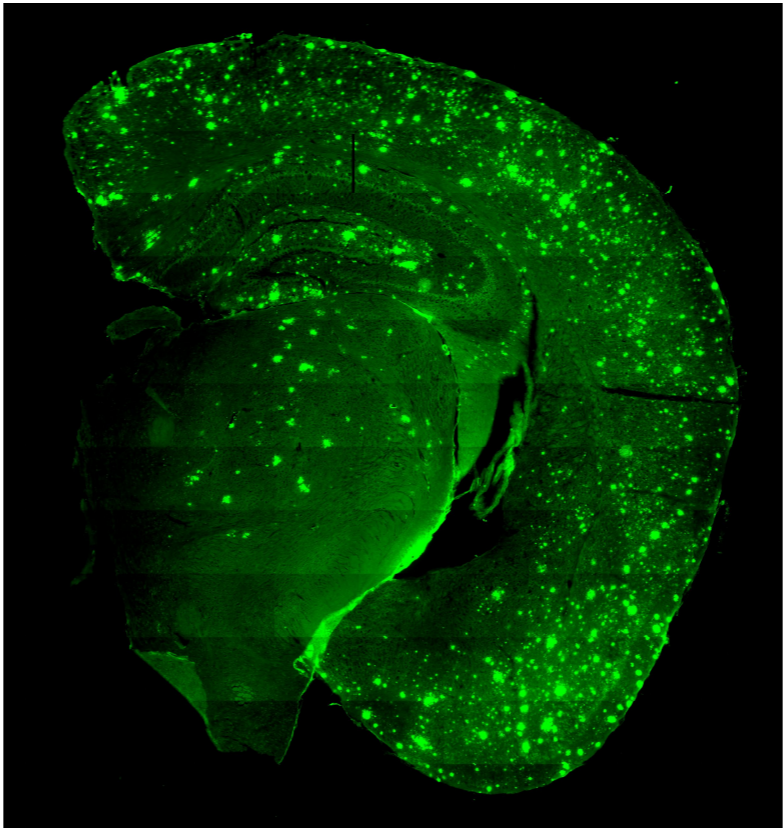

APP/PS1 SS 6E10

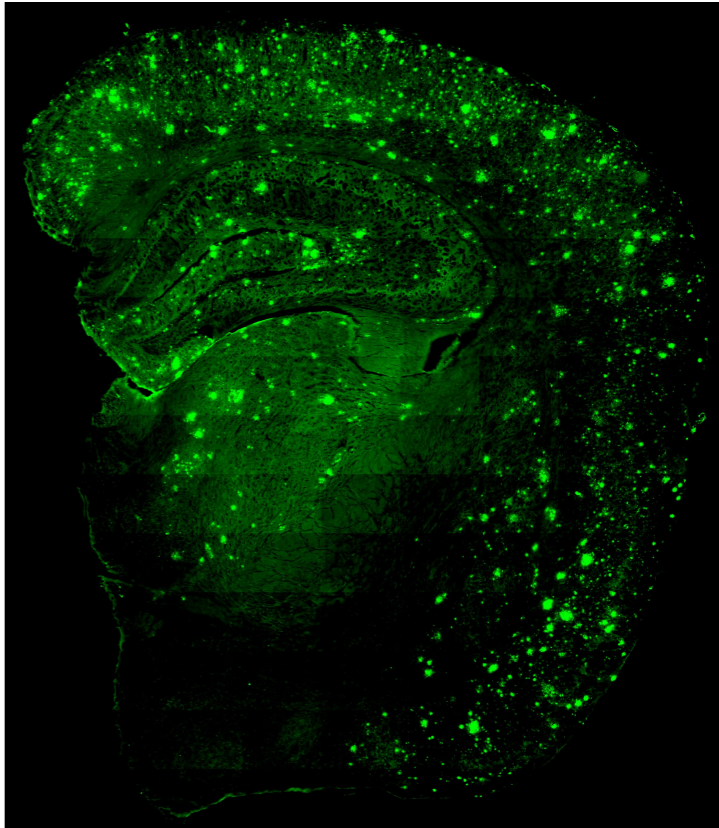

WT Veh GFAP/6E10

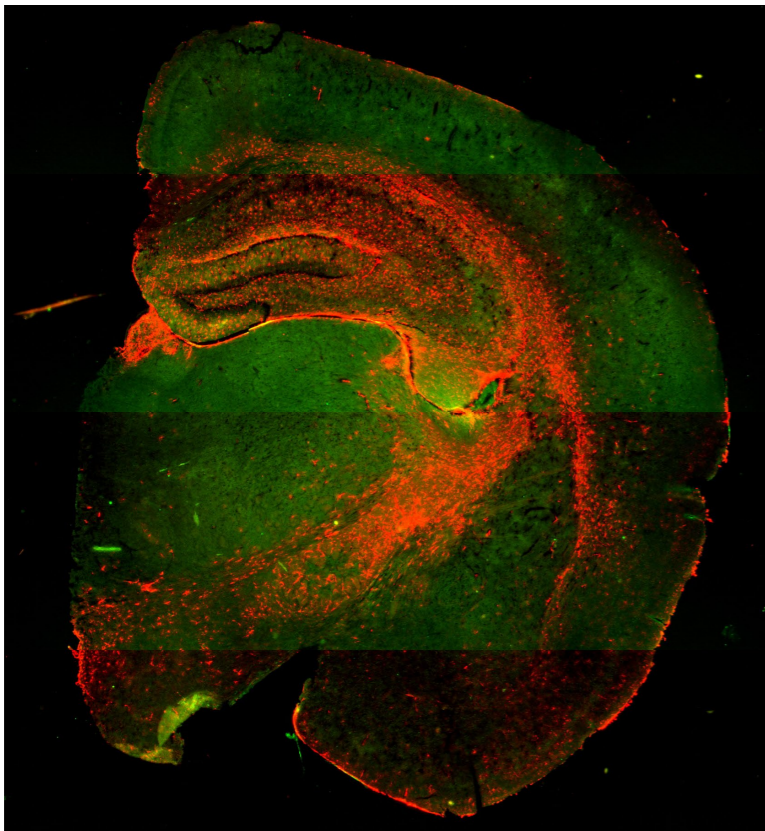

APP/PS1 Veh GFAP/6E10

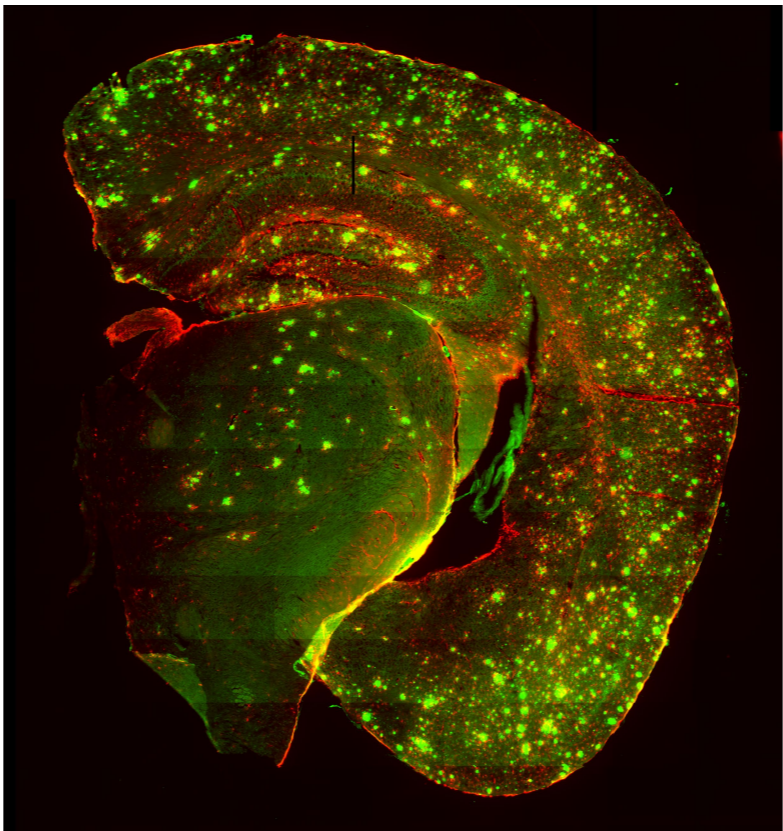

APP/PS1 SS GFAP/6E10

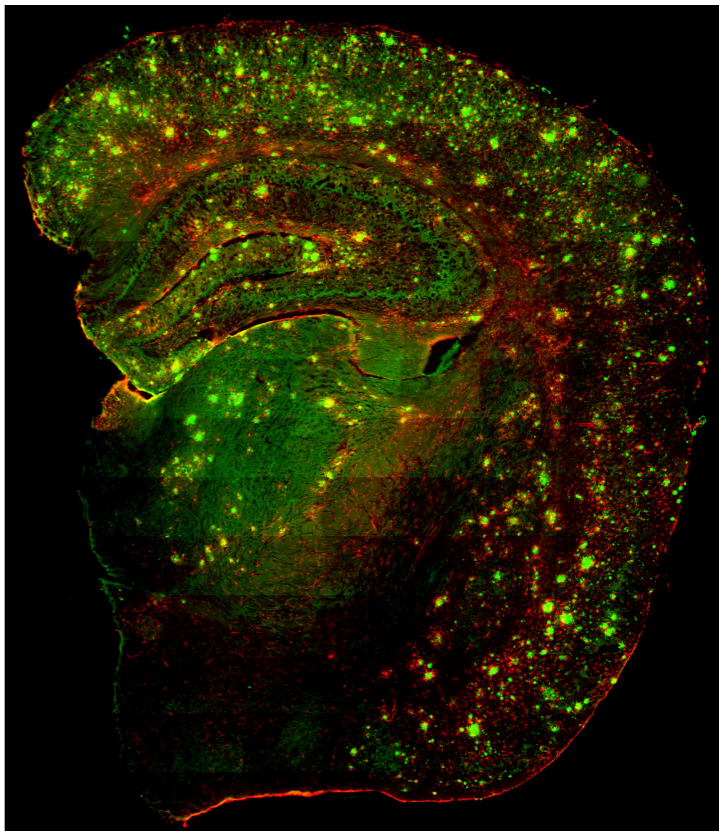

Supplement: S1 File — (PDF) [file pone.0237035.s001.pdf]
