# Supplementary material for: Correction: Smart Soup, a Traditional Chinese Medicine Formula, Ameliorates Amyloid Pathology and Related Cognitive Deficits
Source: PLoS One. 2020 Aug 3;15(8):e0237035. doi: 10.1371/journal.pone.0237035 (PMC7398536; doi:10.1371/journal.pone.0237035)

Figure 2F original images

WT Veh hippocampus

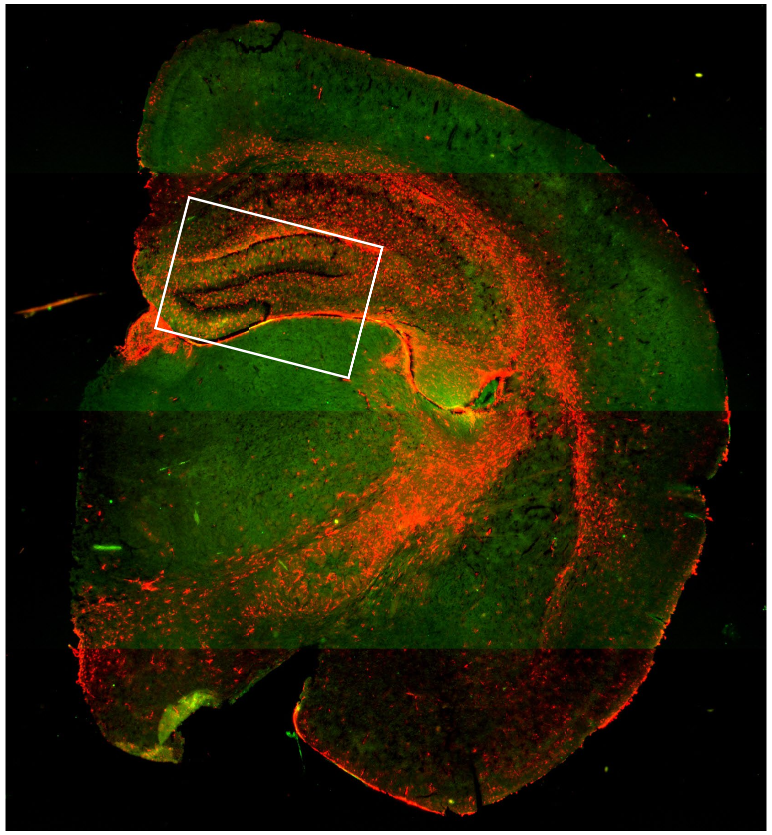

APP/PS1 Veh hippocampus

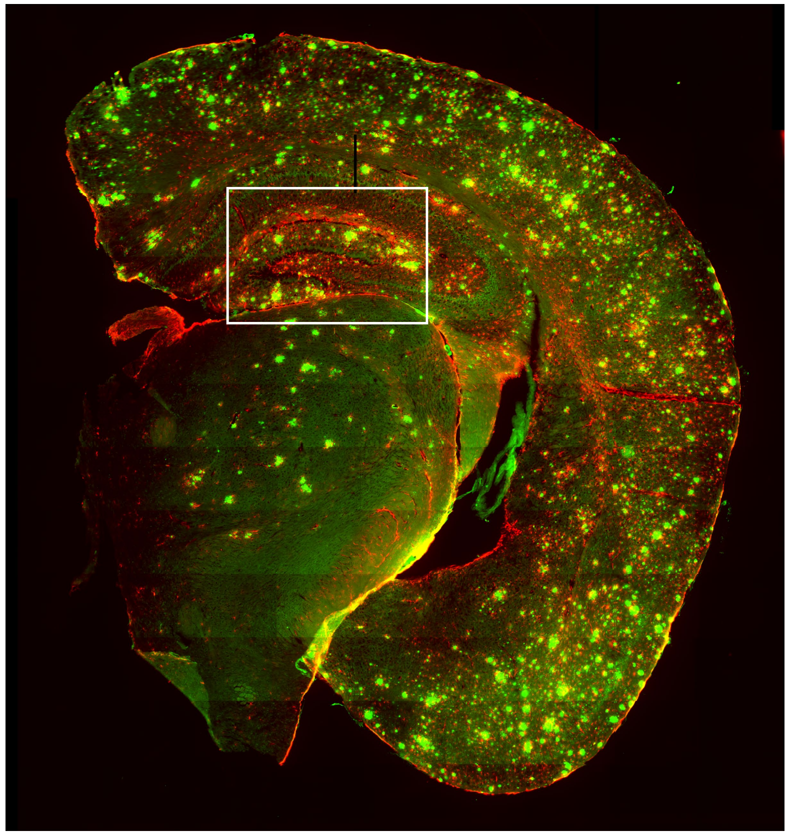

APP/PS1 SS hippocampus

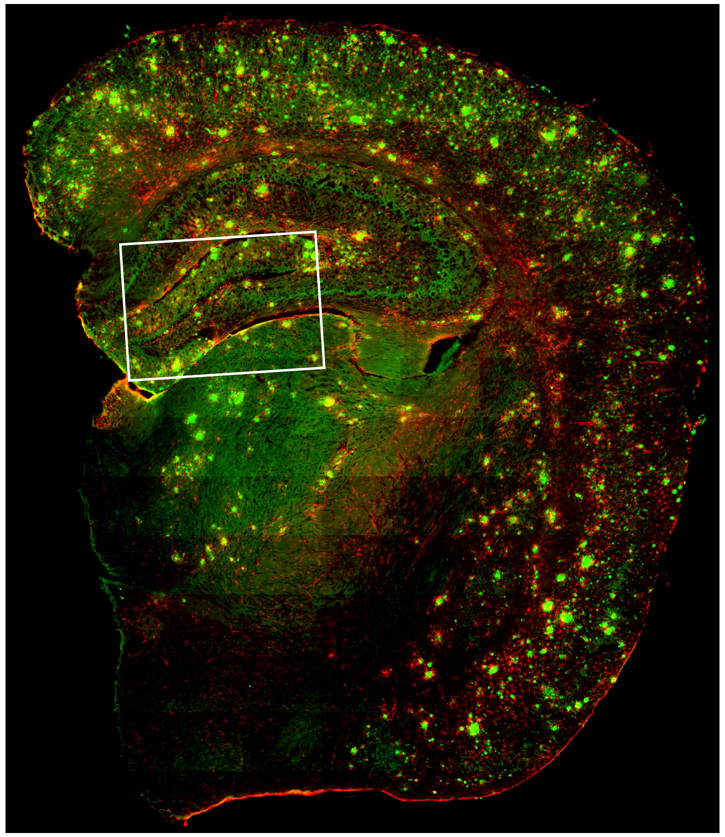

WT Veh cortex

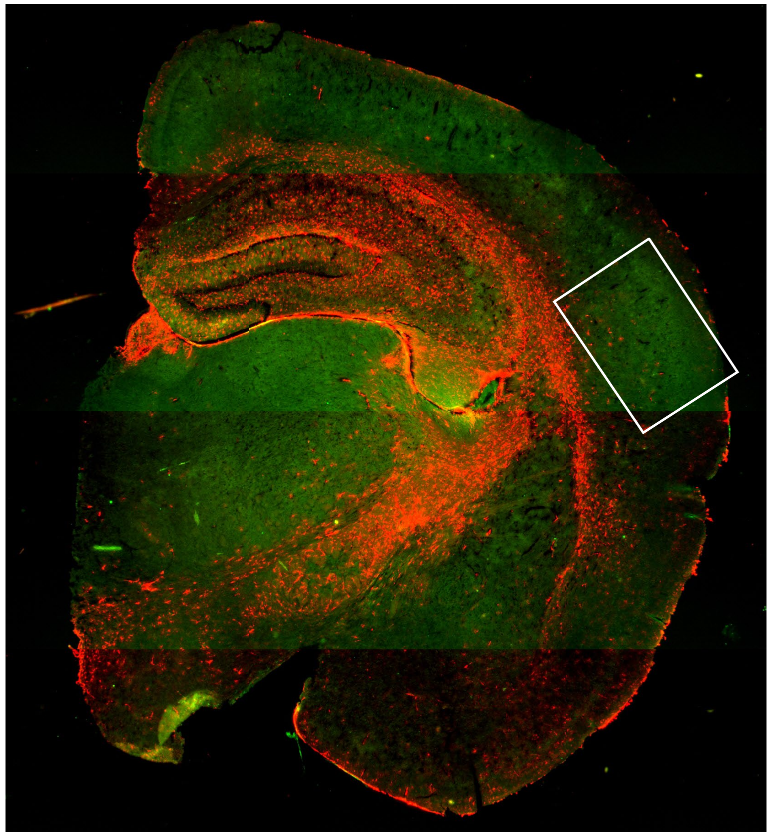

APP/PS1 Veh cortex

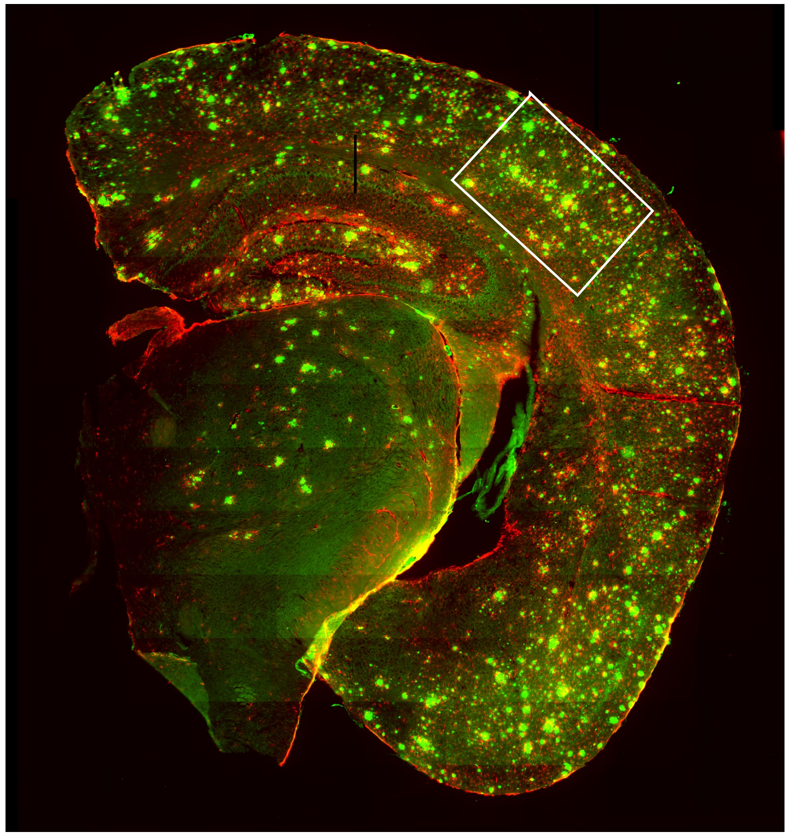

APP/PS1 SS cortex

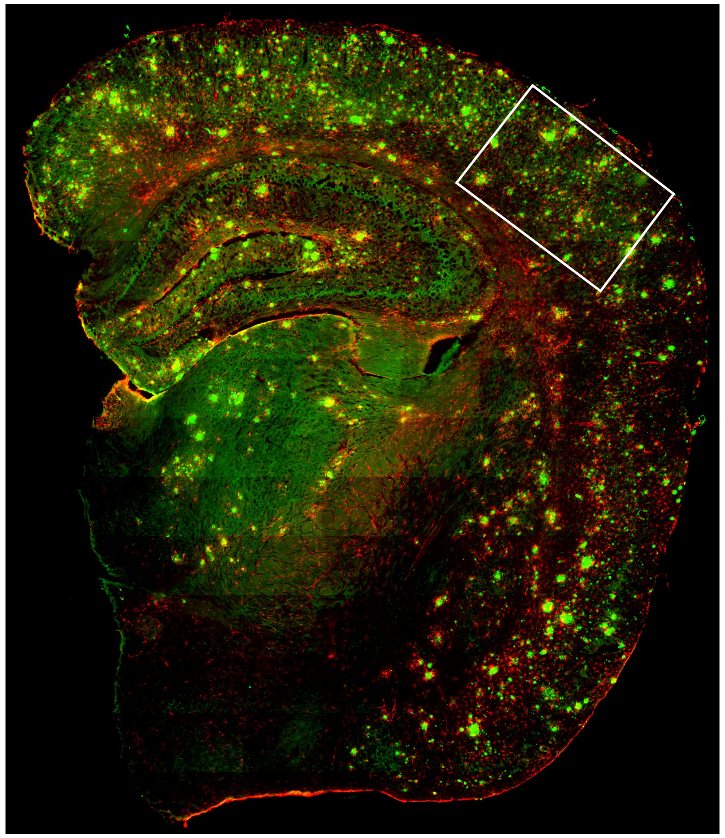

Supplement: S2 File — (PDF) [file pone.0237035.s002.pdf]
