# Supplementary material for: Correction: Smart Soup, a Traditional Chinese Medicine Formula, Ameliorates Amyloid Pathology and Related Cognitive Deficits
Source: PLoS One. 2020 Aug 3;15(8):e0237035. doi: 10.1371/journal.pone.0237035 (PMC7398536; doi:10.1371/journal.pone.0237035)

Figure 2I original images

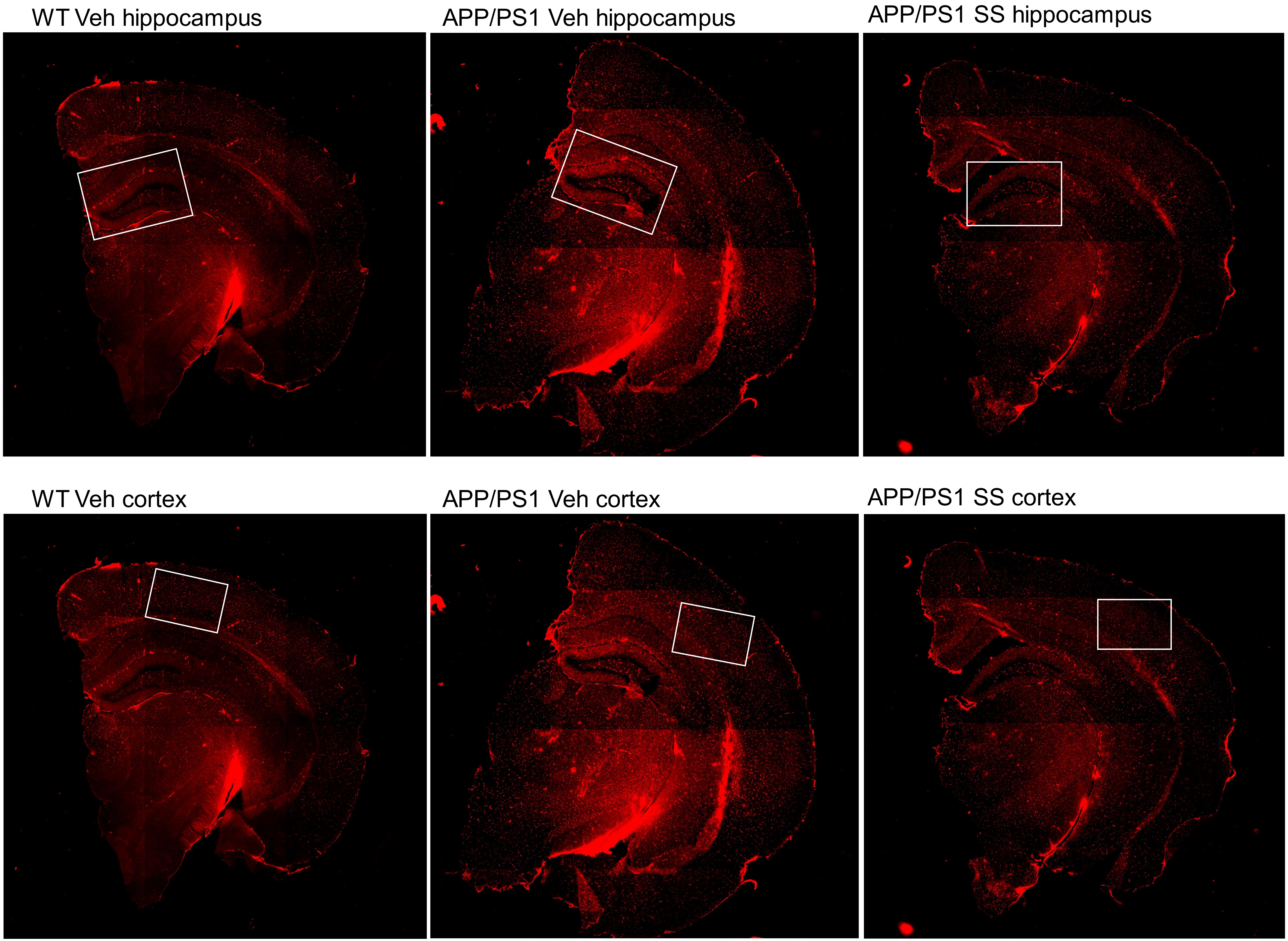

Supplement: S3 File — (PDF) [file pone.0237035.s003.pdf]
